# Controllable Anchoring of Graphitic Carbon Nitride on MnO_2_ Nanoarchitectures for Oxygen Evolution Electrocatalysis

**DOI:** 10.1021/acsami.3c09363

**Published:** 2023-09-28

**Authors:** Mattia Benedet, Andrea Gallo, Chiara Maccato, Gian Andrea Rizzi, Davide Barreca, Oleg I. Lebedev, Evgeny Modin, Ruairi McGlynn, Davide Mariotti, Alberto Gasparotto

**Affiliations:** † Department of Chemical Sciences, Padova University and INSTM, 35131 Padova, Italy; ‡ CNR-ICMATE and INSTM, Department of Chemical Sciences, Padova University, 35131 Padova, Italy; § Laboratoire CRISMAT, UMR 6508 CNRS/ENSICAEN/UCBN, 14050 Caen Cedex 4, France; ∥ CIC nanoGUNE BRTA, Donostia, 20018 San Sebastian, Spain; ⊥ School of Engineering, Ulster University, 2-24 York Street, Belfast BT15 1AP, Northern Ireland

**Keywords:** MnO_2_, nanoarchitectures, graphitic carbon nitride, plasma-enhanced chemical vapor deposition, electrophoretic deposition, oxygen evolution reaction

## Abstract

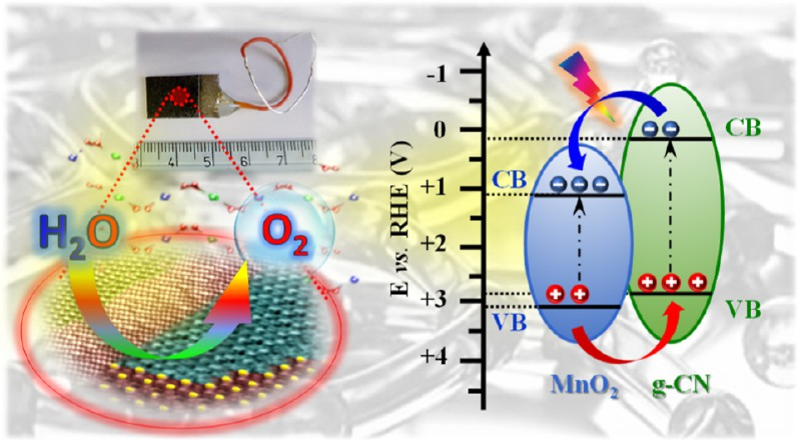

The design and fabrication of eco-friendly and cost-effective (photo)electrocatalysts for the oxygen evolution reaction (OER) is a key research goal for a proper management of water splitting to address the global energy crisis. In this work, we focus on the preparation of supported MnO_2_/graphitic carbon nitride (g-CN) OER (photo)electrocatalysts by means of a novel preparation strategy. The proposed route consists of the plasma enhanced-chemical vapor deposition (PE-CVD) of MnO_2_ nanoarchitectures on porous Ni scaffolds, the anchoring of controllable g-CN amounts by an amenable electrophoretic deposition (EPD) process, and the ultimate thermal treatment in air. The inherent method versatility and flexibility afforded defective MnO_2_/g-CN nanoarchitectures, featuring a g-CN content and nano-organization tunable as a function of EPD duration and the used carbon nitride precursor. Such a modulation had a direct influence on OER functional performances, which, for the best composite system, corresponded to an overpotential of 430 mV at 10 mA/cm^2^, a Tafel slope of ≈70 mV/dec, and a turnover frequency of 6.52 × 10^–3^ s^–1^, accompanied by a very good time stability. The present outcomes, comparing favorably with previous results on analogous systems, were rationalized on the basis of the formation of type-II MnO_2_/g-CN heterojunctions, and yield valuable insights into this class of green (photo)electrocatalysts for end uses in solar-to-fuel conversion and water treatment.

## Introduction

Water splitting, eventually activated by largely available and intrinsically renewable solar light, has been hailed as an extremely attractive route for the carbon-neutral production of green hydrogen (H_2_), paving the way to a new sustainable energy infrastructure in the near future.^1–7^ In general, H_2_O electrolysis involves the hydrogen evolution reaction at the cathode of an electrochemical cell and the oxygen evolution reaction (OER) at the anode, the latter being the overall process bottleneck due to its sluggish kinetics.^4,8–13^ Since large-scale applications of the actual state-of-the-art OER catalysts based on RuO_2_ and IrO_2_^14–16^ are prevented by various issues, encompassing their high cost, insufficient long-term stability, and scarcity,^4,8,10,11^ numerous efforts are being dedicated to alternative eco-friendly electrocatalysts, highly required in the framework of improved sustainability.^9,10,12,13,17^

In this context, graphitic carbon nitride (g-CN), a polymeric metal-free semiconductor, has drawn an ever-growing attention.^2,3,18–22^ This remarkable interest can be traced back to its low cost, nontoxicity, flexible two-dimensional (2D) structure, chemical/thermal stability, tunable defectivity, and appropriate band gap to harvest vis radiation (*E*_G_ ≈ 2.7 eV).^2,8,23–36^ In spite of these advantages, g-CN suffers from low surface area, low electrical conductivity, limited active site availability, and fast recombination of photogenerated charge carriers.^1,5,6,20,26,37–39^ Among the various routes adopted to improve g-CN performances,^19–22,27,29,37^ the controlled construction of heterojunctions between g-CN and a suitable semiconducting partner can yield a more efficient harvesting of solar light and an improved separation of photoproduced electrons and holes.^5,21,23,26,39–41^ Amid the different candidates, MnO_2_, featuring environmental friendliness, earth abundance, and low cost, is an attractive choice,^4,10,12^ thanks to the band structure matching with g-CN one and the narrower band gap (*E*_G_ ≈ 2.0 eV), enabling, in turn, to extend sunlight utilization.^9,27,28,42,43^

So far, MnO_2_ + g-CN composites have been prepared by liquid-phase routes for applications as supercapacitors^33,34,44^ and (photo)catalysts for CO_2_ reduction,^36,37^ toxic product degradation,^18,28,30,31,43^ H_2_ generation,^3,5,6,26,40^ and oxidation reactions of industrial interest.^39,45^ In a different way, the available studies on MnO_2_/g-CN OER electrocatalysts^8,23,42^ (or MnO_2_ composites with C tubes/dots/fibers^1,4,9,11,13,17^) are more scarce. Furthermore, most of the available studies have focused on powdered systems, either as such^1,3,5,18,27,30,32,37,40,41,45^ or mixed with binders/additives to yield pastes immobilized on supports,^8–10,33,42,44^ whereas a primary requirement for real-world applications is the direct growth of active materials onto suitable substrates.^15,24,25,46,47^

The OER activity of the target systems is directly affected by their composition, structure, morphology, and defect content,^4,5,11–13,16,41,45^ as well as by the used support backbone.^8,46^ In particular, the growth of nanosystems on porous and electrically conductive scaffolds, such as Ni foams (NFs),^16,33,34,44^ can favorably yield 3D nanoarchitectures featuring an enriched content of reaction sites, a large active area, and plentiful pathways for ion/charge carrier diffusion.^46–48^ As a matter of fact, further research progress in this direction is highly demanded for the development of green (photo)electrocatalysts endowed with attractive service life and performances.

**Figure 1:**
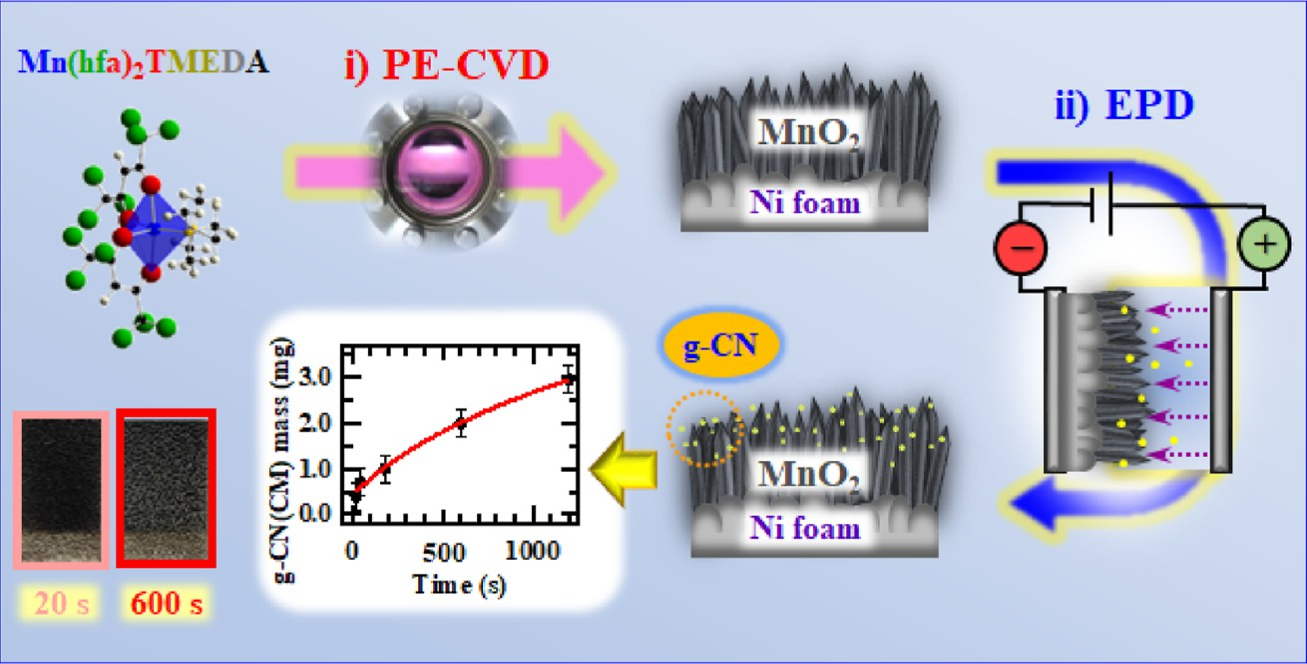
Schematic illustration of the fabrication procedure adopted for MnO_2_-based electrodes functionalized with carbon nitride through: (i) PE-CVD of MnO_2_ on Ni foams; (ii) EPD of g-CN on the developed oxide nanoarchitectures. The bottom panel displays the deposited mass of g-CN(CM) (see the text) as a function of EPD duration (center), as well as the images of two representative deposits (left).

Herein, we report on the fabrication of supported MnO_2_ + g-CN nanocomposites by an original preparation strategy, which, to our knowledge, has never been proposed to date for the preparation of such systems. After the growth of MnO_2_ nanoarchitectures on Ni foam substrates by means of plasma enhanced-chemical vapor deposition (PE-CVD), the anchoring of graphitic carbon nitride is carried out by electrophoretic deposition (EPD), as sketched in Figure 1. Both PE-CVD and EPD techniques are highly versatile for the production of functional nanomaterials with tailored features. Specifically, PE-CVD benefits from the peculiar characteristics of cold plasmas, enabling MnO_2_ growth under conditions far from thermodynamic equilibrium and yielding its efficient dispersion into the used porous substrates.^47,49^ Furthermore, the continuous plasma bombardment of the growing material yields an efficient MnO_2_/Ni foam interfacial contact and tailored surface reactivity.^47,50^ The latter feature, along with the high surface-to-volume ratio of the obtained MnO_2_ deposits, is a favorable issue for the subsequent g-CN anchoring, taking advantage of the EPD flexibility in the processing of exfoliated micro/nanosheets.^25^ Thanks to these particular features, the present approach leads to a high density of MnO_2_/g-CN heterojunctions, favorably influencing electrochemical performances.^47,49^ In this regard, EPD was carried out using two diverse kinds of g-CN with variable defect content and different active areas,^2,7,19,25^ to elucidate the interrelations between the characteristics of carbon nitride and the electrocatalytic activity of the resulting MnO_2_ + g-CN composites.

The prepared systems contain tetragonal β-MnO_2_, the equilibrium polymorph of manganese(IV) oxide,^46^ different from the majority of studies on OER that have been performed on the α phase^8,9,11,26,42^ or amorphous MnO_2_.^23^ The developed nanoarchitectures feature a close MnO_2_/g-CN contact, an open area morphology, and a tunable defect content, resulting in an attractive OER activity. Specifically, the best system yields an overpotential (η) of 430 mV vs the reversible hydrogen electrode (RHE) at a current density of 10 mA/cm^2^ and a Tafel slope of ≈70 mV/dec, which, to our knowledge, are among the best values reported to date for MnO_2_ + g-CN OER catalysts in alkaline solutions. The attractive modular characteristics of the present green materials open the door to their practical use under real-world conditions.

## Experimental Section

### Material Synthesis

MnO_2_ nanoarchitectures were prepared by PE-CVD from electronic-grade Ar/O_2_ plasmas using a custom-built two-electrode apparatus equipped with a 13.56 MHz radio frequency (RF) generator. In each deposition, precleaned^46^ Ni foam substrates (lateral size = 1.2 cm × 2.0 cm) were fixed on the grounded electrode. Based on previously reported results,^46^ growth processes were carried out using the following experimental settings: RF-power = 20 W; deposition temperature = 300 °C; total Ar flow rate = 65 standard cubic centimeters per minute (sccm); total O_2_ flow rate = 5 sccm; duration = 3 h; total pressure = 1.0 mbar.

Anchoring of graphitic carbon nitride on MnO_2_ was performed by EPD from two kinds of carbon nitride powders, i.e., g-CN(M) and g-CN(CM) (where M = melamine and CM = melamine + cyanuric acid, indicating the used precursors; see also the Supporting Information, § S-1.1 and Figures S1 and S2). Depositions were performed from freshly prepared suspensions of g-CN and I_2_ in acetone,^51^ using carbon paper and Ni foam-supported MnO_2_ as anode and cathode, respectively, (see also Supporting Information, § S-1.1). Each composite specimen was finally subjected to annealing at 400 °C for 1 h in air (heating rate = 20 °C/min). The content of g-CN in the final composite systems was modulated as a function of EPD duration [from 20 to 600 s for the systems derived from g-CN(CM)]. For comparison purposes, g-CN(M)-containing composites were prepared by using an optimized deposition time of 180 s. In the following, the target samples are denoted as MnO_2_–X–Y, where X = M or CM and Y = deposition time (s). Bare manganese dioxide (MnO_2_) or g-CN specimens (the latter denoted as X–Y) were also fabricated and characterized.

### Chemicophysical Investigation

A Zeiss SUPRA 40 VP instrument equipped with an INCA x-act PentaFET Precision spectrometer was used for field emission-scanning electron microscopy (FE-SEM) and energy-dispersive X-ray spectroscopy (EDXS) analyses, at primary beam acceleration voltages between 10 and 20 kV. The average aggregate dimensions were evaluated using the ImageJ software through a statistical image analysis.^52^ Transmission electron microscopy (TEM), selected area electron diffraction (SAED), high angle annular dark field-scanning TEM (HAADF-STEM) analyses, and STEM-EDXS mapping were carried out using an aberration double-corrected cold FEG JEM ARM200F microscope operated at 200 kV and equipped with a CENTURIO large angle EDX detector, ORIUS Gatan camera, and Quantum GIF. X-ray photoelectron spectroscopy (XPS) analyses were performed using a ThermoFisher ESCALAB 250XI+ apparatus, with a monochromatized Al Kα X-ray source (*h*ν = 1486.6 eV). Binding energy (BE) values were corrected for charging by assigning a BE of 284.8 eV to the adventitious C 1s signal (component **C**_**0**_ in Figure 6a,b below). Atomic percentages (at.%) were computed by peak area integration using ThermoFisher sensitivity factors. Peak fitting was carried out by XPSPEAK software using Gaussian–Lorentzian sum functions. Photoluminescence (PL) spectra for Ni-foam-supported materials were collected in the 300–700 nm wavelength range using an FLS 1000 fluorimeter (Edinburgh Instruments). The following settings were used: excitation wavelength/bandwidth = 280/13 nm; emission bandwidth = 7 nm.

### Electrochemical Tests

OER performances were tested in freshly prepared 0.1 mol/L KOH (pH = 12.9) solutions with an electrochemical workstation (Autolab PGSTAT204 potentiostat/galvanostat) equipped with a three-electrode setup. A Pt coil, Hg/HgO (MMO), and NF-supported materials were used as counter, reference, and working electrodes, respectively. To prevent wetting of the electrical contact by the electrolyte due to capillary phenomena, a nickel-coated copper wire was carefully welded on the edge of the Ni foam, and the substrate/wire contact was subsequently coated with epoxy resin.

Linear sweep voltammetry (LSV) traces were recorded at a fixed scan rate of 5 mV/s. The onset potential was calculated as the one necessary to reach a current density of 0.02 mA/cm^2^.^53^ Tafel slopes were determined by analyzing the plots of potential vs log(current density).^12,24,46^ Applied bias photon-to-current efficiency (ABPE) curves were obtained by the following equation^23,24,31^
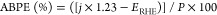
1where *j* (mA/cm^2^) and *P* are the photocurrent density at the potential *E*_RHE_ and the incident light power density (100 mW/cm^2^), respectively.

Additional details and data on material synthesis and characterization are reported in the Supporting Information.

## Results and Discussion

### Material Characterization

Functionalization of PE-CVD MnO_2_ deposits was performed *via* EPD of carbon nitride for different durations, yielding homogeneous samples (Figure 1, left bottom panel). In this study, efforts were dedicated to investigating the influence of EPD duration on the chemicophysical and functional characteristics of the obtained composite systems. As observed in Figure 1 (center bottom panel), varying solely the EPD duration enabled accurate control of the mass of the anchored g-CN even for a very short deposition time, an important issue in modulating material features depending on this parameter.

**Figure 2:**
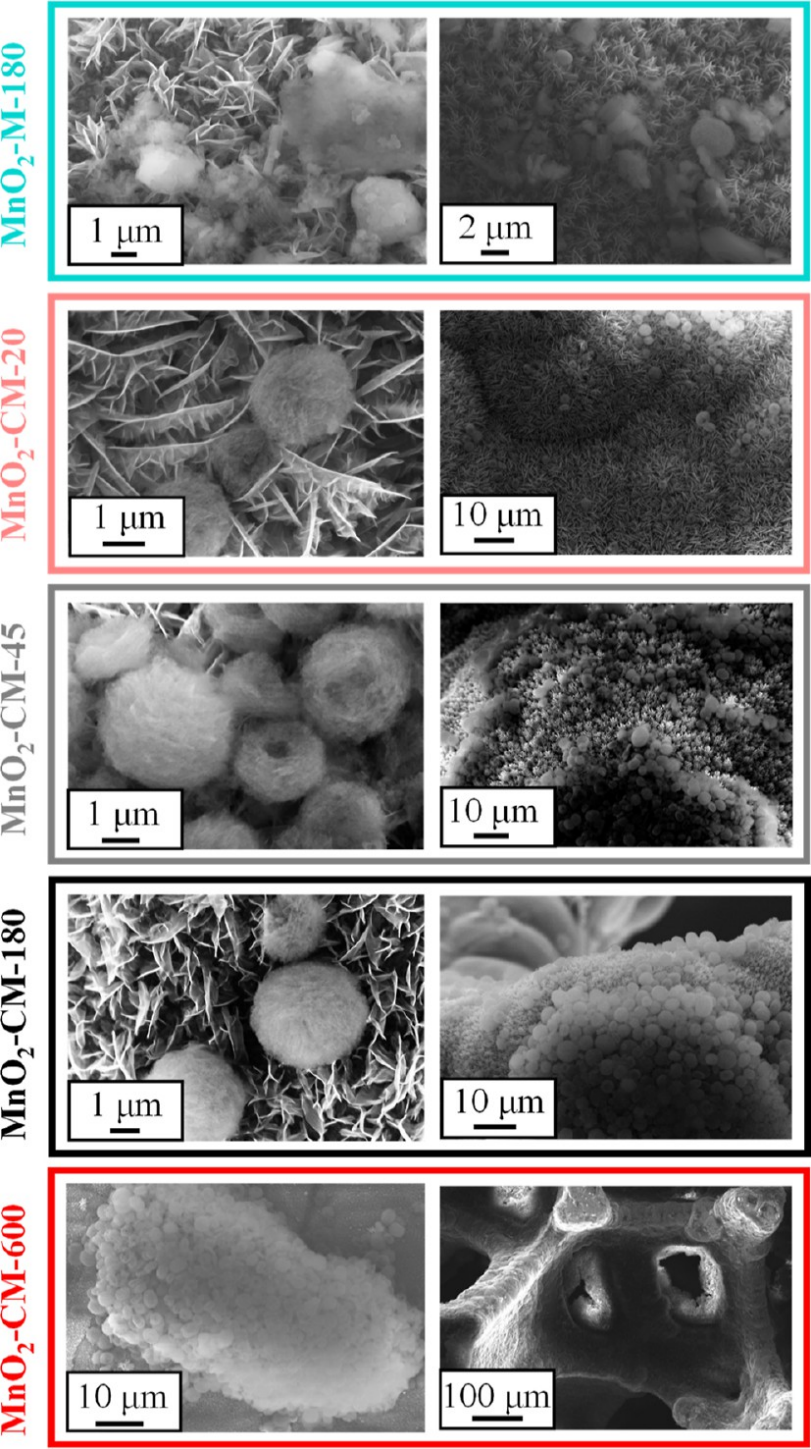
Representative FE-SEM images of MnO_2_ specimens functionalized with g-CN(M) and g-CN(CM).

The system morphology was analyzed by FE-SEM. As can be observed in Figure S3, bare MnO_2_ was dominated by the occurrence of hierarchical 3D *flower-like* architectures resulting from the assembly of 2D flake-like structures. The obtained deposits, evenly covering the underlying Ni foam supports, were characterized by an open morphology and a high active area, allowing enhanced contact with the reaction medium, a key feature for OER applications.^8,11,46,48^ After EPD for moderate times, the oxide matrix showed no remarkable modifications (Figure 2), evidencing that the proposed g-CN anchoring procedure enabled avoiding alterations of the original MnO_2_ morphology. Nonetheless, g-CN nano-organization was appreciably influenced by the EPD duration and the nature of carbon nitride precursor powders. In the case of specimen MnO_2_–M-180, g-CN(M) was characterized by aggregates featuring an irregular shape, with dimensions comprised between 0.5 and 10 μm. In a different way, g-CN(CM)-derived specimens presented spherical-like carbon nitride particles with a narrower size distribution (1–3 μm), resulting from the assembly of exfoliated sheets. As shown by an inspection of Figure 2, a higher EPD process duration resulted not only in an increased amount (in agreement with the mass increase observed in Figure 1, center bottom panel) but also in a different spatial distribution of g-CN(CM). Upon going from MnO_2_–CM-20 to MnO_2_–CM-45, more effective coverage of MnO_2_ by g-CN aggregates was detected, and g-CN particles were still well dispersed onto the underlying MnO_2_. These morphological features evidenced the occurrence of a close contact between MnO_2_ and g-CN(CM), exerting in turn a favorable influence on the ultimate OER activity of composite systems. Nevertheless, a further increase in EPD duration (specimen MnO_2_–CM-180) resulted in an irregular spatial distribution of g-CN, whose globular aggregates completely covered the underlying MnO_2_ in some sample regions (see also the EDXS results presented in Figure S4). This phenomenon was even more evident in the sample MnO_2_–CM-600, for which the functionalization with carbon nitride yielded even a partial occlusion of Ni foam pores. These characteristics detrimentally affected the resulting electrochemical activity, as explained below.

**Figure 3:**
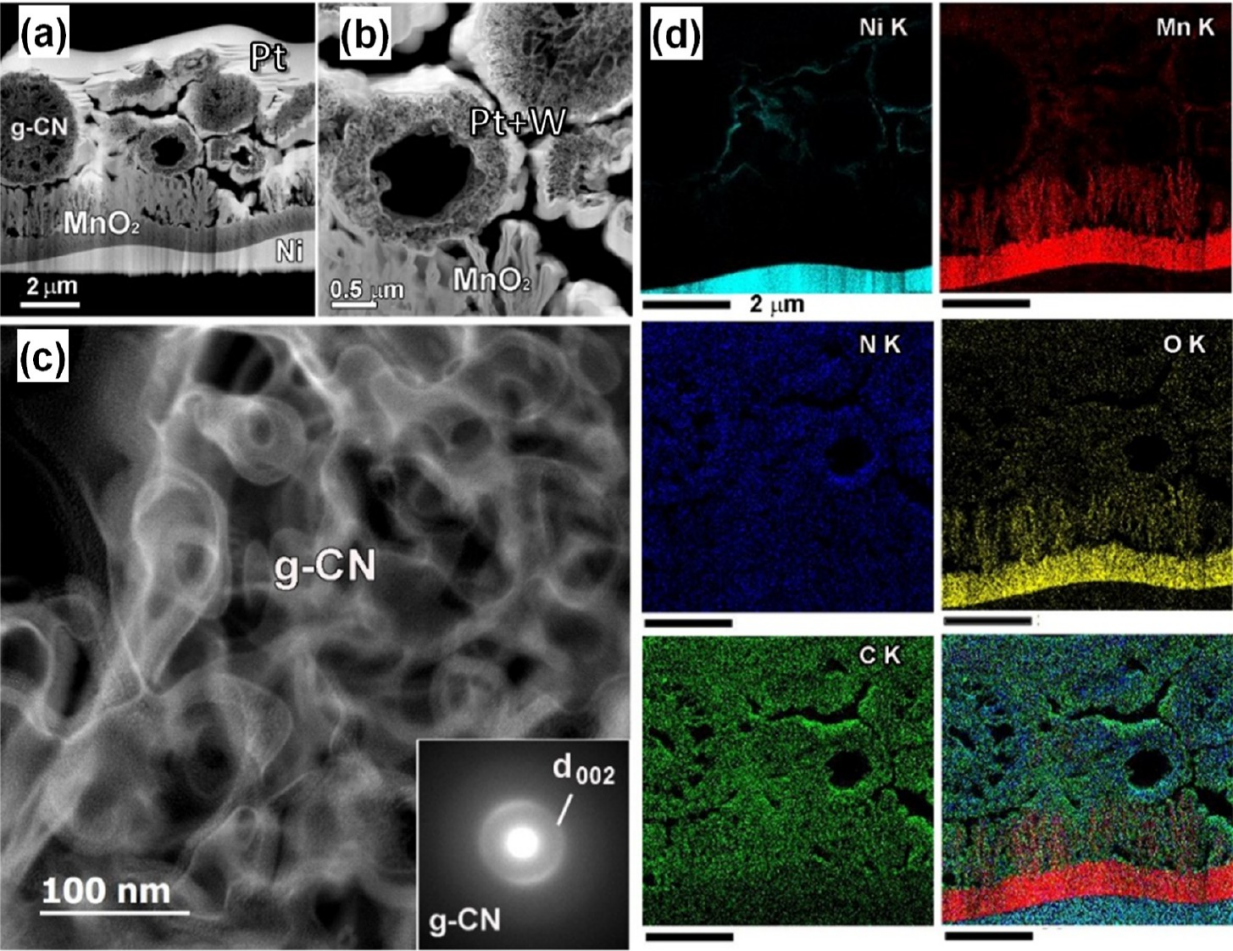
(a) Cross-sectional HAADF-STEM overview and (b,c) magnified images of MnO_2_–CM-45. The SAED pattern recorded on g-CN aggregates is displayed as an inset in (c). (d) Corresponding STEM-EDXS chemical maps for Ni Kα, Mn Kα, N Kα, O Kα, and C Kα and an overlaid image. Pt and W in parts (a) and (b) are due to sample preparation prior to analysis.

**Figure 4:**
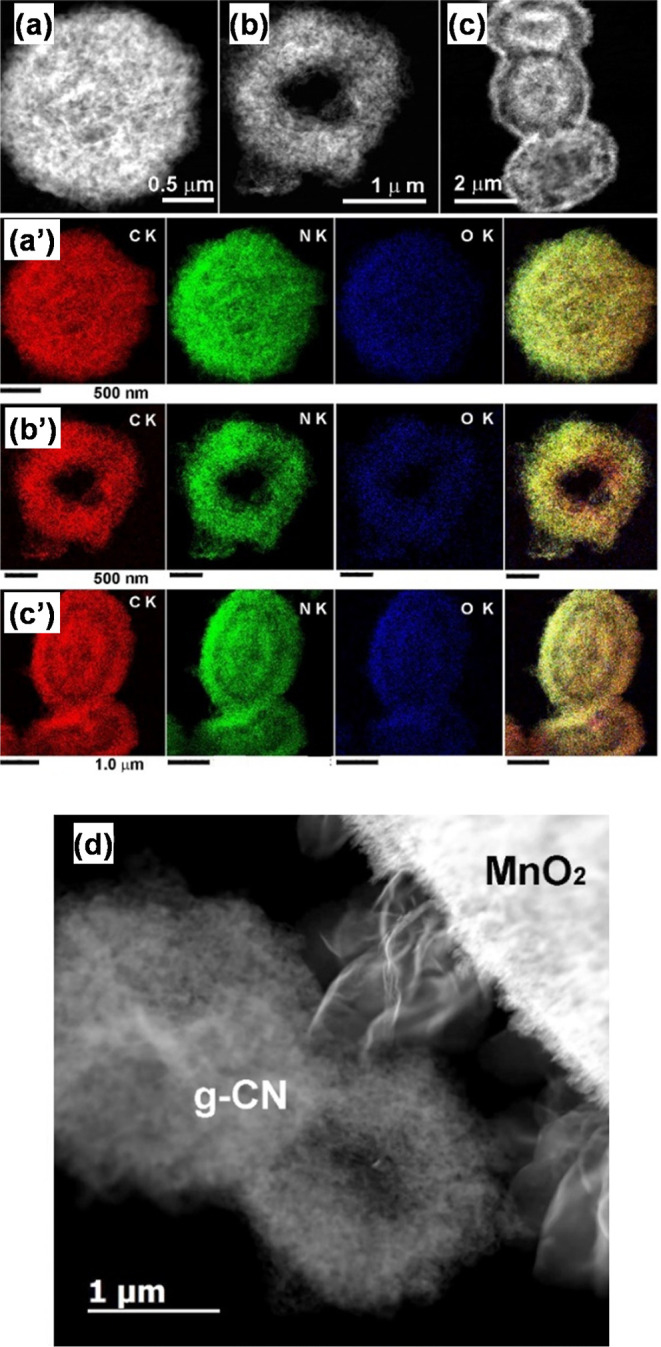
Low-magnification HAADF-STEM images of different g-CN(CM) particles: (a) spherical mesoporous; (b) donut-type; (c) spherical double-layered. The bottom panel displays the corresponding STEM-EDXS elemental maps for C Kα, N Kα, and O Kα, and the overlaid color images for (a′) spherical mesoporous; (b′) donut-type; (c′) spherical double-layered particles. (d) HAADF-STEM image of the g-CN(CM)/MnO_2_ interface.

Preliminary Raman spectroscopy and X-ray diffraction (XRD) analyses (see Figures S5 and S6 and related observations) evidenced the obtainment of composite systems, in which phase-pure β-MnO_2_ and g-CN maintained their chemical identity. To attain a deeper insight into the system nano-organization, with particular regard to carbon nitride spatial distribution and to MnO_2_/g-CN interfaces, an advanced TEM and EDXS investigation was performed on selected samples. Figure 3 displays representative HAADF-STEM images and STEM-EDXS chemical maps for specimen MnO_2_–CM-45. The obtained results revealed the occurrence of MnO_2_ dendritic grains forming a relatively dense film on the Ni foam substrate [thickness = (1.0 ± 0.2) μm]. Above this layer, MnO_2_ featured a flake-like morphology resulting in a more porous deposit [thickness = (1.0 ± 0.1) μm], covered by randomly distributed g-CN spherical aggregates (dimensions between 1 and 3 μm). The SAED pattern of the latter (Figure 3c, inset) displayed a faint (002) g-CN ring [interplanar spacing, *d*_002_ = (0.32 ± 0.05) nm].^25^ A thorough analysis revealed that g-CN presented different 3D porous structures: spherical mesoporous, donut-type, and spherical double-layered (see HAADF-STEM images and STEM-EDXS maps in Figure 4). Regardless of their morphology, all particles were formed by assembled g-CN flakes. The latter were evenly distributed within mesoporous spherical particles, whereas donut-type systems presented a porous shell structure surrounding a hollow internal cavity. Conversely, spherical double-layered systems consisted of a g-CN core region comprised between two denser shells with a thickness of (400 ± 30) nm, separated by a less compact layer [thickness = (200 ± 30) nm]. Tilting experiments in the HAADF-STEM mode (Figure S7) confirmed the 3D morphology of the observed g-CN aggregates. As a matter of fact, similar hierarchical structures can advantageously increase the content of surface active sites, favoring the separation of photogenerated charge carriers and thus boosting material performances.^54–56^ As can be observed in Figure 4d, g-CN particles are well connected to flake-like MnO_2_, yielding an intermixed interfacial region that can promote charge transport processes.

**Figure 5:**
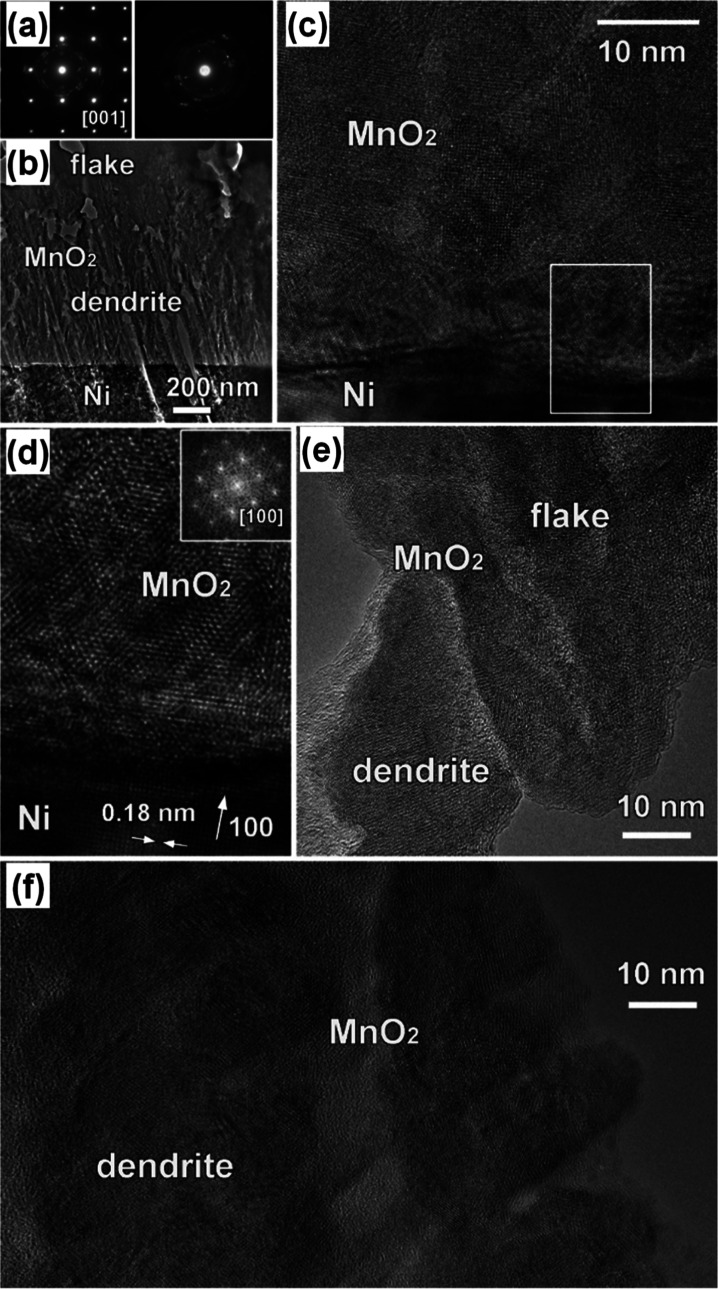
TEM analysis for the MnO_2_-M-180 specimen. (a) SAED patterns taken from a [001]-oriented MnO_2_ dendritic grain (left) and from the flake-like MnO_2_ region (right), marked in the HAADF-STEM image reported in (b). Both patterns can be indexed based on the tetragonal β-MnO_2_ structure (*P*4_2_/*mnm* space group; *a* = 4.39 Å, *c* = 2.87 Å).^57,58^ (c) HRTEM image of the MnO_2_/Ni foam interface. (d) Higher magnification micrograph of the area framed by the white box in (c). Inset: Fourier transform pattern, confirming the [100] orientation of epitaxially grown MnO_2_ grains. (e) HRTEM image of the dendrite/flake MnO_2_ interface. (f) HRTEM image of the upper part of the MnO_2_ dendrite region in (b).

Figure 5 displays the TEM characterization results of sample MnO_2_–M-180. As can be observed, after a critical thickness of 600 nm, the MnO_2_ deposit starts switching from a dendrite-type to a flake-type growth. According to the collected SAED patterns and HRTEM images, both dendrite- and flake-like MnO_2_ particles possess a tetragonal structure. Figure 5c,d shows evidence that dendritic grains present a near-epitaxial relationship with the Ni foam substrate. In a different way, no epitaxial relationship between dendritic and flake-type MnO_2_ particles, that showed a random orientation, was detected (see Figure 5e).

**Figure 6:**
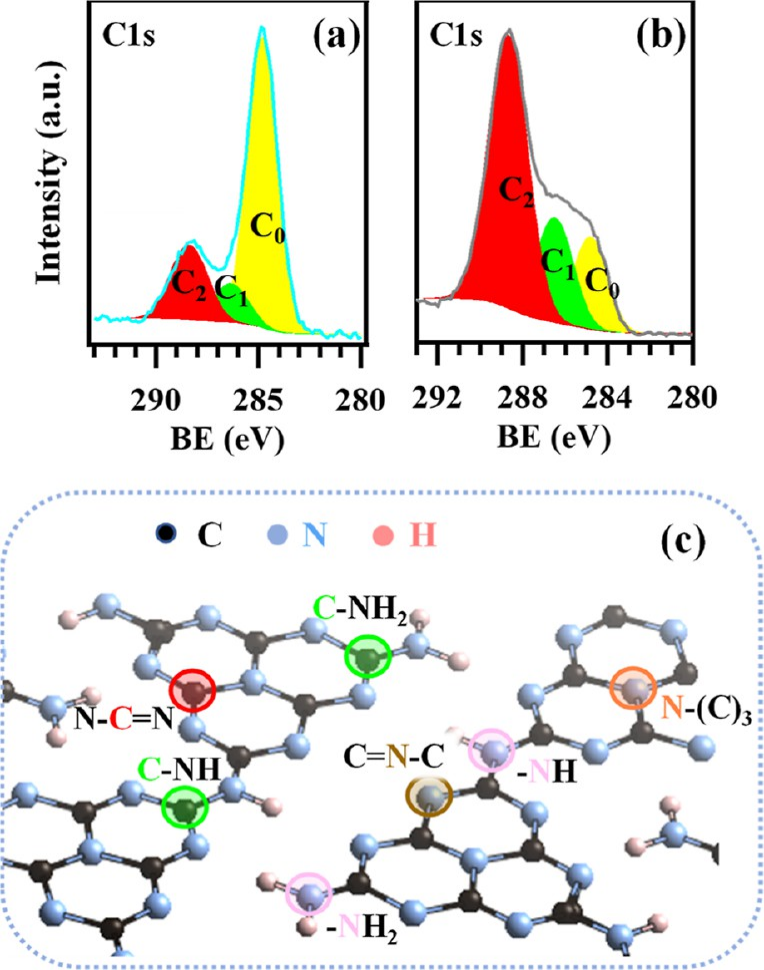
C 1s photopeaks for representative MnO_2_ + g-CN samples: (a) MnO_2_–M-180; (b) MnO_2_–CM-45. (c) Sketch of graphitic carbon nitride structure,^61^ in which the various C and N sites are marked in the left and right image, respectively. Color codes identifying nonequivalent carbon atoms are the same ones used for the different fitting components in panels (a,b).

**Figure 7:**
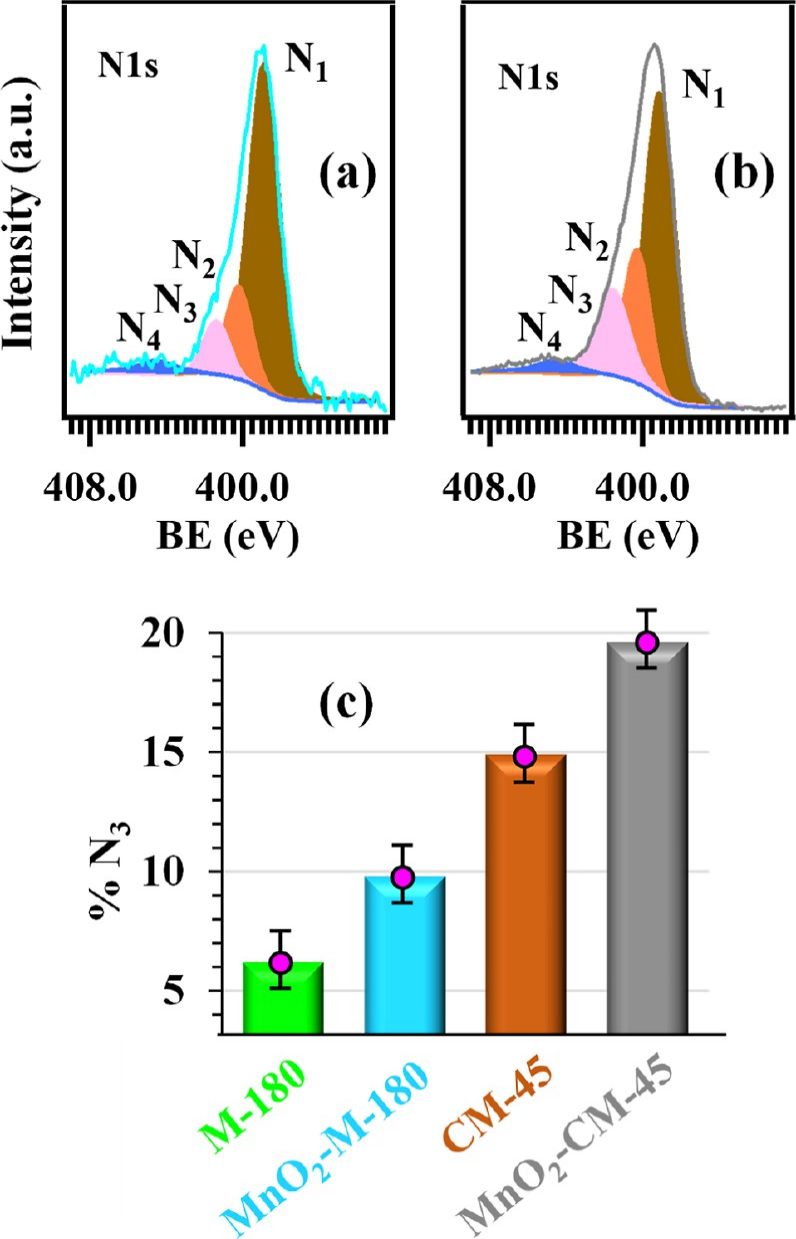
N 1s photopeaks for representative MnO_2_ + g-CN samples: (a) MnO_2_–M-180; (b) MnO_2_–CM-45. Color codes for the different fitting components identifying nonequivalent nitrogen atoms are defined in Figure 6c. (c) Percentage contribution of the **N**_**3**_ component to the overall N 1s signal for the analyzed specimens.

Material chemical composition was analyzed by XPS on representative samples. For bare manganese dioxide and composite materials, the O/Mn atomic percentage (at.%) ratio values were very close to 2.0, as expected for MnO_2_. Conversely, N/C atomic percentage ratios (1.0–1.1) were lower than the stoichiometric value for g-C_3_N_4_ (1.3), suggesting that the target systems were nitrogen-deficient, a feature favorably impacting their photoactivity.^38^ C 1s signals resulted from the contribution of three different bands (Table S1 and Figure 6a,b; see Figure 6c for the detailed attribution): **C**_**0**_, due to adventitious carbon contamination, at 284.8 eV;^32,45^**C**_**1**_, related to C bonded to amino groups (C-NH_*x*_; *x* = 1, 2) on g-CN heptazine ring edges;^38,59,60^**C**_**2**_, attributed to carbon in N–C = N moieties of g-CN aromatic rings.^3,7,34,38,40^ For composite systems (see also Table S1), the **C**_**1**_ component could be also related to C–O bonds between g-CN and MnO_2_,^1,3,31,32,40,43^ in line with O 1s peak analysis results (see Figure S9 and Table S5). This C–O bonding suggests the formation of a solid MnO_2_/g-CN linkage,^43^ a favorable feature promoting electron–hole separation.^37^ Upon passing from bare g-CN specimens to the corresponding composites, **C**_**1**_ and **C**_**2**_ components underwent a BE increase^6,32,44^ (see Table S1), higher upon going from CM-45 vs MnO_2_–CM-45 (0.4 eV) in comparison to the one occurring in MnO_2_–M-180 with respect to M-180 (0.2 eV). These results suggested the occurrence of a g-CN → MnO_2_ electron flow,^18^ consistent with the formation of g-CN/MnO_2_ heterojunctions. N 1s photopeaks were fitted with four components (Figure 7a,b): **N**_**1**_, the main one, attributed to C = N–C bicoordinated nitrogen centers;^8,37,61^**N**_**2**_, due to tertiary N–(C)_3_ atoms in g-CN;^7,35,38,59^**N**_**3**_, assigned to uncondensed NH_*x*_ moieties;^19,32,35,59^**N**_**4**_, related to π–π*-electron excitations.^7,30,34,45^ The contribution of component **N**_**3**_ to the overall N 1s signal underwent a nearly 2-fold increase upon going from specimen M-180 to CM-45 and from sample MnO_2_–M-180 to MnO_2_–CM-45 (Table S2 and Figure 7c), highlighting that CM-derived systems were characterized by a higher content of terminal -NH_*x*_ groups, i.e., by a lower condensation degree. In line with the C 1s case,^18,23^ even the BEs of N 1s components underwent an increase in going from pure nitrides to composite samples (see Table S2), supporting the presence of the aforementioned MnO_2_/g-CN electronic interplay.

**Figure 8:**
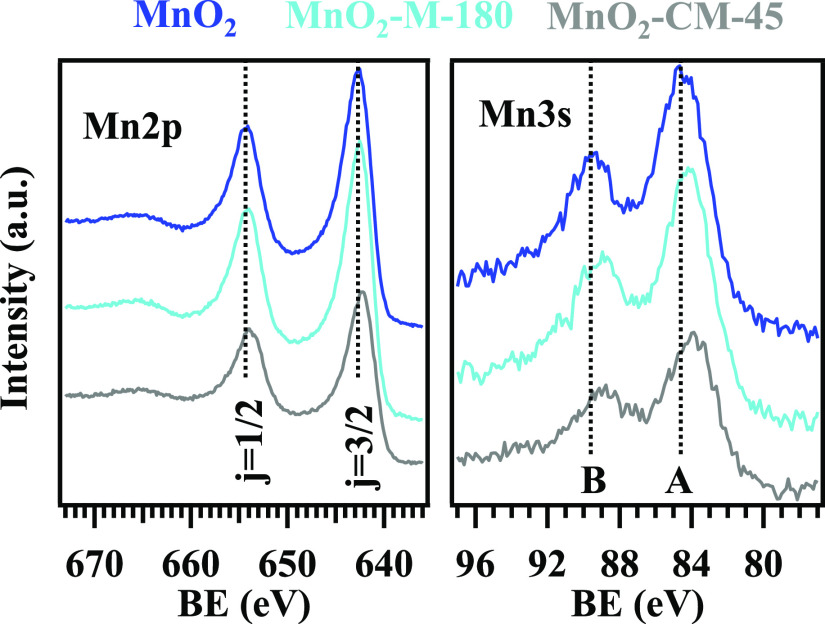
Mn 2p (left) and Mn 3s (right) photopeaks for the MnO_2_ and MnO_2_ + g-CN samples. In both cases, the component positions for bare MnO_2_ are marked with dashed lines. In the right panel, the two Mn 3s components generated by multiplet splitting are marked by **A** and **B**.

Manganese photoelectron peaks are displayed in Figure 8. For bare MnO_2_, the obtainment of manganese(IV) oxide was confirmed by Mn 2p signal shape and position [Table S3; BE(Mn 2p_3/2_) = 642.6 eV;^15,30,44,62^ spin–orbit splitting (SOS) = 11.6 eV^1,41,43,44^], as well as by Mn 3s multiplet splitting separation^45,62^ (Table S4). For composite materials, Mn 2p and Mn 3s energy positions underwent a red shift in comparison to MnO_2_ (−0.2 eV for MnO_2_–M-180 and −0.4 eV for MnO_2_–CM-45; Tables S3 and S4). This phenomenon, in accordance with previously reported data for MnO_2_/g-CN materials,^6,18,23,43,44^ confirmed that g-CN and MnO_2_ were directly coupled in the fabricated composite systems,^34^ and that a built-in electric field was formed at the g-CN/MnO_2_ interface.^41^ The occurrence of the latter promoted a g-CN → MnO_2_ electron transfer, a phenomenon which, on the basis of the measured BE shifts, turned out to be more marked for MnO_2_–CM-45 than for MnO_2_–M-180. The g-CN/MnO_2_ interaction was further proven by photoluminescence measurements on bare MnO_2_, CM-45, and MnO_2_–CM-45. The recorded spectra (Figure S10) revealed that MnO_2_ did not yield any appreciable emission. In the case of g-CN-containing samples, a better charge separation, corresponding to luminescence quenching, was observed for specimen MnO_2_–CM-45 in comparison to CM-45. This phenomenon is accompanied by a red shift of the emission maximum (≈5 nm for MnO_2_–CM-45 vs CM-45), indicating a favorable electronic interaction between MnO_2_ and g-CN, suppressing the recombination of photogenerated charge carriers.^63^ Basing also on FE-SEM and TEM outcomes, this result was related to the exfoliated morphology of the g-CN(CM) complex, enabling shortening of the charge carrier diffusion length and promoting g-CN → MnO_2_ electron transfer,^29^ in line with XPS results (see above).

### Electrochemical Characterization

**Figure 9:**
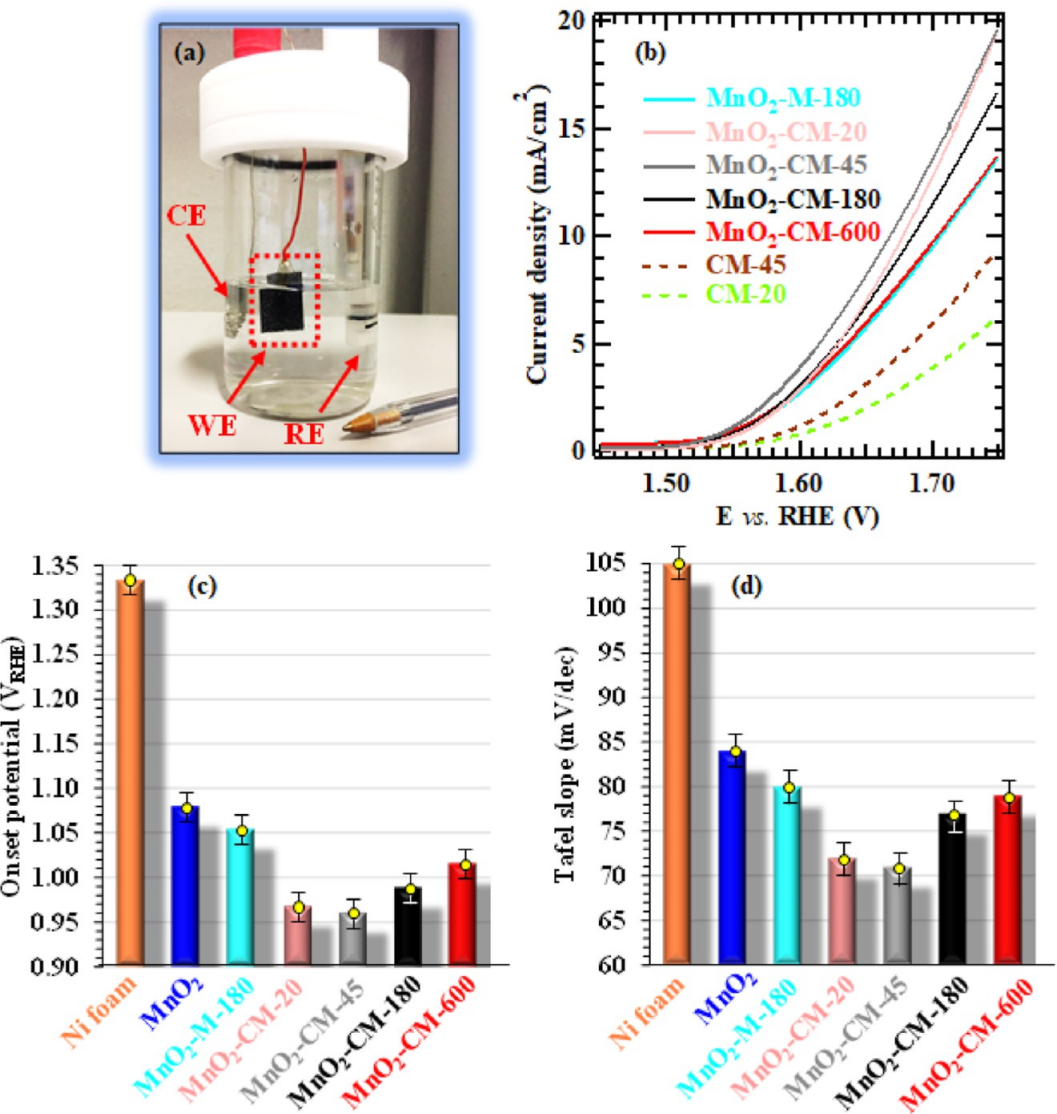
(a) Photograph of the cell used for electrochemical tests (CE = counter-electrode; WE = working electrode; RE = reference electrode). The anodic photocurrent proves the n-type semiconducting nature of the target materials.^14^ (b) LSV curves recorded under irradiation, (c) onset potentials, and (d) Tafel slopes for the indicated specimens.

The catalytic activity of the obtained systems (Figure 9a) was investigated in alkaline media. The LSV curves recorded under irradiation for single-phase and composite materials (Figure 9b) evidenced a net current density increase at potentials >1.50 V vs RHE. The appreciable dark currents at high bias values (Figure S11) indicate that the target materials are reasonable OER catalysts.^14^ As a general trend, LSV data under illumination indicated that the current densities yielded by composite systems were higher than the ones measured not only for the Ni foam substrate, but also for bare g-CN and MnO_2_ samples, provided that an optimal carbon nitride amount was anchored on the underlying manganese oxide (compare Figures 9b and S12; see also onset potential and Tafel slope values (Figure 9c,d). The extrapolated onset potentials (Figure 9c) were directly influenced by both the used g-CN precursor (as shown by the values for MnO_2_–M-180 and MnO_2_–CM-180, the latter yielding a higher activity) and the EPD duration (as shown by the data for the g-CN(CM)-containing composites). Based on the above-described characterization results, a similar phenomenon was related to the concurrence of (a) the higher exfoliation of CM-derived systems, resulting in an enhanced active area and, in turn, in better contact with the reaction medium; (b) the improved MnO_2_/g-CN linkage in CM-derived systems, and their higher defect content, as evidenced by XPS results. In particular, defects resulting from uncondensed –NH_*x*_ groups can suppress charge carrier recombination, favorably boosting the system’s electrocatalytic activity.^24,25^ Furthermore, a detailed analysis of Figure 9c revealed, for CM-derived specimens, a marked dependence of onset potentials on EPD process times, with the best-performing system (MnO_2_–CM-45) yielding a potential drop of ≈0.37 V in comparison to that of bare MnO_2_. Based on FE-SEM outcomes (see Figures 2 and S3 and related observations), these effects can be attributed to the fact that longer EPD durations yield an irregular and excessive accumulation of carbon nitride aggregates on MnO_2_, thus hindering hole transport to the g-CN surface and suppressing the advantages brought about by MnO_2_/g-CN junctions. In a different way, a too low carbon nitride content (as for specimen MnO_2_–CM-20) reduces MnO_2_/g-CN heterocontact density, limiting the benefits yielded by MnO_2_/g-CN coupling and thus compromising the resulting functional performances.

Information about the OER kinetics was gained from the Tafel slopes (Figure 9d). The obtained results indicate that g-CN anchoring on MnO_2_ promoted a Tafel slope decrease according to the trend: Ni foam ≫ MnO_2_ > MnO_2_–M-180 > MnO_2_–CM-600 > MnO_2_–CM-180 > MnO_2_–CM-20 > MnO_2_–CM-45 (the latter sample yielding a reduction of 34 mV/dec with respect to MnO_2_). As can be observed, the obtained slopes presented the same trend of onset potentials (compare Figure 9c,d), with lower values for CM-derived materials and, in particular, for specimen MnO_2_–CM-45. Since lower Tafel slopes are indicative of an improved reaction kinetics, i.e., of a more efficient O_2_ evolution at the anode,^9,11,12^ these results further validate the positive influence exerted by g-CN(CM) on OER activity. It is worthwhile to observe that the functional performances of the present systems in terms of current densities at 1.65 V vs RHE, as well as Tafel slope and overpotential values, compared favorably not only with some of the previously reported electrocatalysts with analogous composition but also with various RuO_2_ and IrO_2_-based benchmark systems (compare data in Table S6 with the ones in Tables S7 and S8). Taken together, these observations demonstrate that the adopted synthetic procedure yielded MnO_2_ + g-CN composites of potential interest for a possible practical implementation.

**Figure 10:**
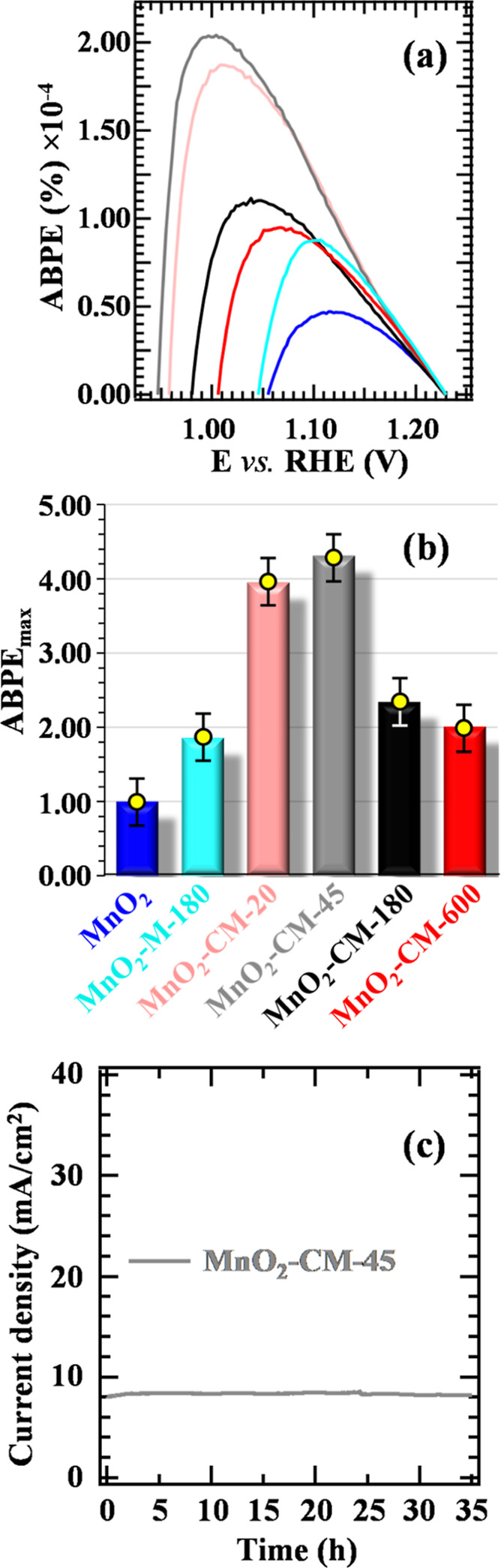
(a) ABPE % curves for bare MnO_2_ and MnO_2_ + g-CN electrodes; color codes are as in (b). (b) Plot of the relative maximum efficiency (ABPE_max_), normalized with respect to bare MnO_2_. (c) Prolonged chronoamperometric (CA) measurements for MnO_2_–CM-45, performed at 1.65 V vs RHE.

The activity order discussed above was in excellent agreement with the ABPE results. As can be observed in Figure 10a, whereas bare MnO_2_ was the worst electrocatalyst, g-CN introduction resulted in an increase of ABPE maximum value (Figure 10b), accompanied also by a favorable curve shaping toward lower bias values (see also Table S6, last column). The best-performing MnO_2_–CM-45 electrocatalyst exhibited a more-than-4-fold ABPE maximum increase in comparison to the pristine MnO_2_ and presented the highest turnover frequency (TOF; Table S6). Altogether, these results indicate that the coupling of MnO_2_ and g-CN(CM) can advantageously boost electrocatalytic performances, provided that a judicious choice of g-CN loading (i.e., of the EPD process duration) is performed.

In view of eventual real-world end uses, a good system service life is a key requirement.^15,17,24^ Preliminary CA measurements conducted for 1 h (Figure S13) showed that all of the target materials exhibited good stability. In order to attain additional information, CA experiments were conducted for up to 35 h for the best-performing electrocatalyst (Figure 10c), highlighting a constant current density throughout the whole experiment. To unambiguously discard the occurrence of material alterations upon aging, the target systems were stored for six months under ambient conditions and periodically subjected to electrochemical tests. The obtained data (Figure S14) revealed only modest current variations, allowing us to rule out any significant degradation or dissolution, as confirmed by *postoperando* XRD and FE-SEM measurements (Figures S15 and S16). These results are of high importance toward an eventual practical utilization, considering also that the present systems are eco-friendly and economically viable.

**Figure 11:**
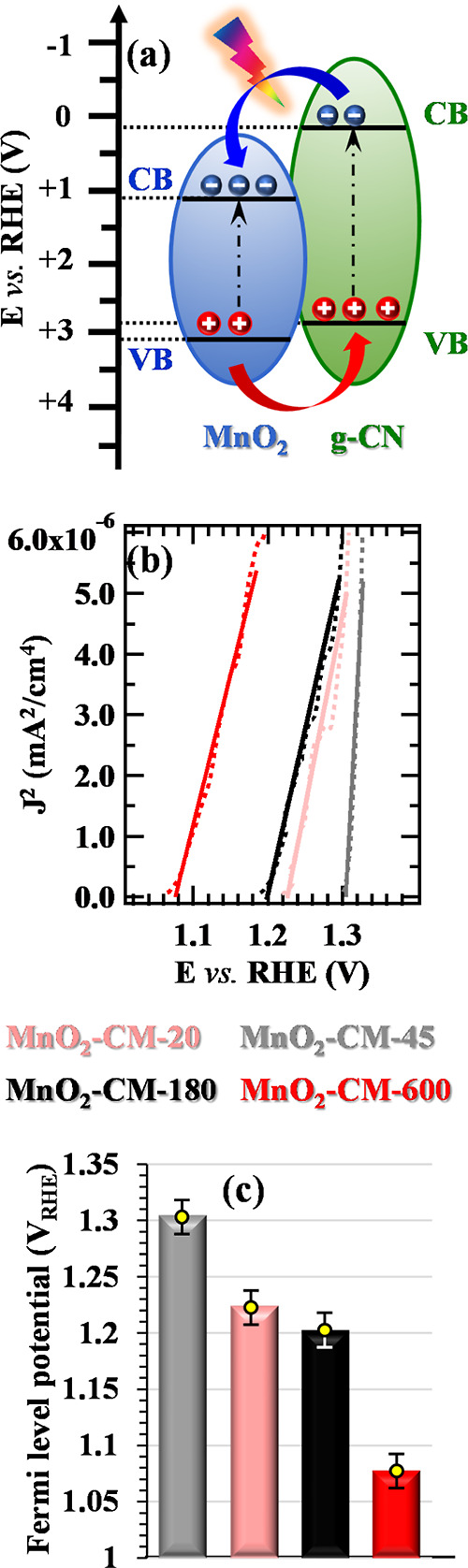
(a) Schematic energy band diagram for MnO_2_/g-CN systems.^68^ Conduction and valence band edges are denoted as CB and VB, respectively. (b) Square photocurrent density (*J*^2^) vs applied bias for MnO_2_ + g-CN(CM) electrodes. Experimental and fitting curves correspond to dashed and continuous lines. (c) Extrapolated Fermi level potentials for the target specimens.

The electrocatalytic activity enhancement promoted by g-CN anchoring on MnO_2_ can be traced back to cooperative morphological, catalytic, and electronic effects. In fact, high-area nanoarchitectures like the present ones feature an enhanced OER activity,^8,46,48^ since they can (i) maximize the contact with the working solution and provide enough room for reactant transport even into the inner system regions; (ii) reduce the diffusion distance of charge carriers from catalytic sites to the Ni foam substrate and, hence, to the external circuit. These conclusions are corroborated by Tafel slope values, indicating that the anchoring of g-CN results in a promotional catalytic effect. A last important contribution is provided by the interfacial electronic interplay between MnO_2_ and g-CN, and, in particular, by the formation of type-II junctions^63,64^ (see Figure 11a). Specifically, upon irradiation and bias application, holes flow from the MnO_2_ valence band to the g-CN one, whereas electrons can be transferred from the g-CN conduction band to the MnO_2_ one. Electrons are then injected into the Ni foam substrate and, subsequently, transported to the cathode, where they are involved in reduction processes leading to H_2_. The most likely OER active sites are pyridinic nitrogen atoms, on which the highest occupied molecular orbital of g-CN is mainly localized.^65,66^ The important influence exerted by MnO_2_/g-CN junctions on the ultimate material activity is further highlighted by electrochemical impedance spectroscopy data collected for bare MnO_2_ and MnO_2_–CM-45 specimens. In fact, the pertaining results (Figure S17) show, under illumination, a more effective charge transfer resistance decrease for sample MnO_2_–CM-45 with respect to that for bare MnO_2_.

The above observations account for the activity increase brought about by composite systems, but how does one explain the different performances of MnO_2_ + g-CN(CM) electrocatalysts obtained using different EPD durations? To attain a deeper insight into this issue, flat band potential values were estimated by the intercept of the square photocurrent density curves with the potential axis (Figure 11b).^14,67^ The Fermi level potentials reported in Figure 11c are the electrode potentials at which no band bending occurs.^67^ An inspection of the obtained results reveals that the Fermi level position is directly dependent on the target electrocatalyst, with an increase according to the order MnO_2_–CM-600 < MnO_2_–CM-180 < MnO_2_–CM-20 < MnO_2_–CM-45. This sequence, in excellent agreement with the above-discussed electrochemical results, explains why MnO_2_–M-45 is the best-performing electrocatalyst among CM-derived ones since a deeper Fermi level energy enables one to achieve the OER reaction onset with a lower electrode polarization.

## Conclusions

In summary, eco-friendly electrocatalysts based on graphitic carbon nitride and MnO_2_ were fabricated by an original preparation strategy, consisting of the PE-CVD of manganese dioxide nanoarchitectures on Ni foams followed by g-CN electrophoretic deposition and final annealing in air. Particular attention was devoted to the anchoring on MnO_2_ of tunable amounts of exfoliated g-CN with a high active area and defect content. A thorough analytical investigation by complementary techniques highlighted the successful obtainment of nanoarchitectures featuring an even g-CN spatial distribution and intimate MnO_2_/g-CN coupling, which is of key importance to benefit from their synergistic interplay in OER electrocatalysis. The best composite system yielded an overpotential of 430 mV to achieve a current density of 10 mA/cm^2^ and a Tafel slope of ≈70 mV/dec, favorably comparing with various previously reported MnO_2_ + g-CN OER catalysts. These performances, accompanied by remarkable material stability, could be rationalized on the basis of the formation of type-II MnO_2_/g-CN heterojunctions and on the different Fermi level positions as a function of g-CN content, tunable solely as a function of the EPD duration.

In perspective, a computational investigation of the target systems will be of importance to attain deeper insight into the mechanism governing their electrocatalytic performances. Furthermore, the present nanocomposites stand as promising platforms for water treatment applications, and the fabrication strategy utilized in this study can be exploited to prepare a variety of multicomponent non-noble electrocatalysts for H_2_O splitting end uses. In this regard, it is worthwhile noting that the presently adopted preparation route is potentially viable for the industrial production of large-area, porous electrode architectures since both PE-CVD and EPD allow an easy immobilization of the active catalyst components over a variety of substrate materials.

## Supporting Information Available

Characterization details; additional IR, optical spectroscopy, SEM, EDXS, XRD, TEM, and XPS results; photoluminescence data; electrochemical data; chronoamperometric measurements; and postoperando XRD and FE-SEM analyses (PDF)
