# Supplementary material for: Controllable Anchoring of Graphitic Carbon Nitride on MnO2 Nanoarchitectures for Oxygen Evolution Electrocatalysis
Source: ACS Appl Mater Interfaces. 2023 Sep 28;15(40):47368–80. doi: 10.1021/acsami.3c09363 (PMC10571007; doi:10.1021/acsami.3c09363)
Supplement: Supplementary file 1 — am3c09363_si_001.pdf [file am3c09363_si_001.pdf]

Supporting Information

Controllable Anchoring of Graphitic Carbon Nitride on MnO_2 Nanoarchitectures for Oxygen Evolution Electrocatalysis

Mattia Benedet,[†,§] *Andrea Gallo,*[†] *Chiara Maccato,*[†,§] *Gian Andrea Rizzi,*[†,§] *Davide Barreca,*[*,§] *Oleg I. Lebedev,*[‖] *Evgeny Modin,*[#] *Ruairi McGlynn,*[⊥] *Davide Mariotti,*[⊥] *and Alberto Gasparotto*[†,§]

[†] Department of Chemical Sciences, Padova University and INSTM, 35131 Padova, Italy

[§] CNR-ICMATE and INSTM, Department of Chemical Sciences, Padova University, 35131 Padova, Italy

[‖] Laboratoire CRISMAT, UMR 6508 CNRS/ENSICAEN/UCBN, 14050 Caen Cedex 4, France

[#] CIC nanoGUNE BRTA, 20018 Donostia, San Sebastian, Spain

[⊥] School of Engineering, Ulster University, 2-24 York Street, Belfast, BT15 1AP Northern Ireland

* Corresponding author; E-mail: davide.barreca@unipd.it.

Table of Contents

	Page
**§ S-1. Additional experimental details**	S3
**§ S-1.1 Synthesis**	S3
**§ S-1.2 Characterization**	S4
**§ S-2. Additional characterization results**	S7
**§ S-2.1 Characterization of g-CN powders**	S7
**§ S-2.2 Characterization of the obtained systems**	S9
**§ S.3. Electrochemical tests and related analyses**	S19
**§ S.4. References**	S28

§ S-1. Additional experimental details

§ S-1.1 Synthesis

Synthesis of MnO_2 deposits. Plasma enhanced-chemical vapor deposition (PE-CVD) of MnO_2 was performed on porous Ni foams (Ni-4753, RECEMAT BV), endowed with a desirable open-pore structure providing excellent mass transport and a large surface per unit area.[1-2] In order to remove the native oxide formed upon air exposure and other eventual undesired impurities,[1-4] Ni foams were pre-cleaned by sonication in dichloroethane (10 min), 3.5 M HCl (10 min) and ethanol (5 min), and finally dried under an Ar stream.

The compound $Mn(hfa)_2TMEDA$ (hfa = 1,1,1,5,5,5-hexafluoro-2,4-pentanedionate; TMEDA = *N,N,N',N'*-tetramethylethylenediamine),[5-6] used as manganese molecular precursor, was heated at 70°C in an external glass vessel, and its vapors were transported into the reaction chamber by an Ar flow [rate = 60 standard cubic centimeters per minute (sccm)] through gas lines maintained at 130°C by means of external tapes. Additional Ar and O_2 flows (rates = 15 sccm and 5 sccm, respectively) were separately introduced into the reactor through independent inlets.

After each deposition, the deposited MnO_2 mass was determined using a Mettler Toledo XS105 DualRange microbalance. The average value obtained for 10 different depositions was (0.55±0.05) mg, indicating a high reproducibility of the adopted experimental conditions.

In this work, two types of carbon nitride powders, involving the use of melamine (M) or cyanuric acid + melamine (CM) as starting precursors, were fabricated to yield systems with different active areas,[7] following slight modifications of previously reported literature procedures.

Synthesis of g-CN(M) powders.[7] 2.0 g of melamine (M – Sigma Aldrich) were finely grinded, dispersed in an alumina crucible, and pre-treated under Ar at 100°C for 30 min (heating rate = 2°C/min) to remove residual moisture. The sample was subsequently annealed under Ar for 2 h at 400°C, to promote melamine condensation to tri-s-triazine, and finally at 550°C for 4 h, to induce the polymerization to carbon nitride. After cooling to room temperature, yellowish powders [g-CN(M)] were obtained.

Synthesis of g-CN(CM) powders.[8] Melamine (M – Sigma Aldrich; 1.0 g) and cyanuric acid (C – Sigma Aldrich; 1.0 g) were dissolved under sonication in 40 mL and 20 mL of dimethylsulfoxide (DMSO), respectively. After heating at 60°C, the C-containing suspension was added dropwise to the M-containing one under vigorous stirring, yielding a white suspension of

the supramolecular C+M complex (CM complex). After 10 min, the suspension was filtered, and the adduct was washed twice with 10 mL of ethanol. The resulting solid was heated under Ar for 3 h at 50°C and, afterwards, for 2.5 h at 500°C, ultimately affording light brown powders [g-CN(CM)].

Electrophoresis of g-CN on MnO_2 deposits. Electrophoretic deposition (EPD) of carbon nitride on Ni foam-supported MnO_2 deposits was carried out following a previous procedure.[9] In particular, g-CN and I_2 (40 and 10 mg, respectively) were finely grinded and suspended in acetone (50 mL). After sonication for 20 min, the suspension was utilized for EPD, using an applied potential of 5 V for g-CN(M) and of 10 V for g-CN(CM). In each process, the suspension was maintained under mild stirring, and an Agilent E3649A bench power supply was adopted to apply a fixed potential difference between the two electrodes. Before each deposition, carbon paper, employed as anode, was subjected to a pre-cleaning treatment with deionized water, isopropyl alcohol and acetone.

Thanks to H^+ ions formed through the following reaction:

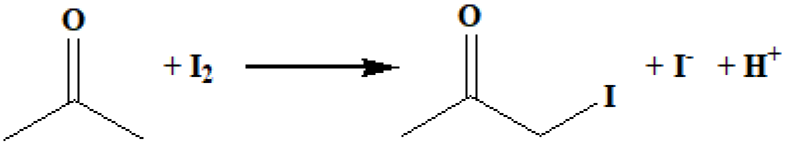

(S1)

g-CN particles acquire a positive charge, which favors their transport towards the cathode (in the present case, Ni foam-supported MnO_2 systems).[10]

§ S-1.2 Characterization

FT-IR spectra were registered on a JASCO 4100 instrument in attenuated total reflectance (ATR) mode (PIKE IRIS ATR). The estimated uncertainty on the reported wavenumbers is ± 4 cm^{-1}.

A Bruker AXS D8 Advance Plus diffractometer equipped with a Göbel mirror and a CuKα X-ray source (λ = 1.54051 Å) powered at 40 kV, 40 mA was used for X-ray diffraction (XRD) measurements. The latter were carried out at the PanLab facility (Department of Chemical Sciences, Padova University) founded by the MIUR Dipartimento di Eccellenza grant "NExuS". The estimated uncertainty on the reported 2θ values is ± 0.1°.

Optical absorption spectra were collected on a Cary 50 (Varian) spectrophotometer (spectral

bandwidth = 1 nm), operating in diffuse reflectance mode. Band gap (E_G) evaluation was performed using the Tauc equation $[f(R)h\nu]^n$ *vs.* $h\nu$, where f(R) is the Kubelka-Munk function and R is the reflectance, assuming the occurrence of allowed and indirect electronic transitions (n = ½).[7,11-13]

Raman spectra were recorded using a ThermoFisher DXR Raman microscope using a 780 nm laser (5 mW) focused on the sample with a 100x objective (Thermo-Fisher Scientific), obtaining a spot size of ≈ 1 μm. The estimated uncertainty on the reported Raman shift values is ± 5 cm^{-1}.

In the preparation of specimens for transmission electron microscopy (TEM) observations, due to the extreme fragility of g-CN nanostructures under ion beam irradiation, Pt and W were deposited to protect carbon nitride aggregates and obtain cross-section lamellaes (including Ni foam substrate, MnO_2 and on-top g-CN) endowed with the adequate electron transparency. For high resolution TEM (HRTEM) investigation, two additional systems were prepared: i) a first one, by focused ion beam (FIB), is the thin lamella containing only Ni foam and MnO_2, used for the analysis of MnO_2 and MnO_2/Ni foam interface; ii) a second specimen, by ultrasonication for 15 min in ethanol and subsequent deposition on a TEM grid. This procedure enabled to obtain separate g-CN nanostructures for HRTEM analyses and tilting experiments.

Electrocatalytic tests were performed both in the dark and upon illumination with a white light source (Philips LUMILEDS LXML-PWN1 0120; 220 lm, 700 mA).

To perform a direct comparison of the electrocatalytic activity for the various specimens, current density (j) values (estimated uncertainty: ± 0.01 mA/cm^2) were obtained by normalizing the measured currents to the geometric electrode area[4,14] (1.2×1.5 cm^2). In fact, BET measurements of the actual surface areas for supported nanosystems like the present ones are not feasible. In such cases, conventional N_2 or Kr physisorption methods are not applicable, due to the extremely low mass of the active material with respect to the supporting substrate, as already reported.[4] On the other hand, scratching of the deposited material from the substrate induces further uncertainties. In addition, material chemico-physical features, along with the inherent uncertainty in the estimation of double layer capacitance and specific capacitance,[15-16] do not enable a consistent estimation of electrochemical active surface area (ECSA).

Potential values *vs.* Hg/HgO (MMO) were converted into the reversible hydrogen electrode (RHE) scale using the following equation:[17]

$$E_{RHE}(V) = E_{MMO}(V) + 0.059\times pH + 0.111 \quad (S2)$$

The overpotential for oxygen evolution (η) was calculated for the different specimens as:[14,17-18]

$$\eta(\mathrm{V}) = \mathrm{E}^{10}_{\mathrm{RHE}}\ (\mathrm{V}) - 1.23 \quad \text{(S3)}$$

where E^{10}_{RHE} (V) is the potential at j = 10 mA/cm^2, and 1.23 is the E^0 value for O_2 evolution, both on the RHE scale.

The electrocatalyst turnover frequency (TOF) was evaluated using the relation:[19-20]

$$\mathrm{TOF} = (j\times A) / (4\times F\times n) \quad \text{(S4)}$$

where j is the current density at a given overpotential (η = 420 mV), A is the geometric electrode area (see above), F is the Faraday constant (96485 C×mol^{-1}), and n are the moles of manganese on the electrode. The estimated uncertainty on the obtained TOF values is ± 0.2×10^{-3} s^{-1}.

Chronoamperometric (CA) analyses were performed at a fixed potential value of 1.65 V *vs.* RHE.

§ S-2. Additional characterization results

§ S-2.1 Characterization of g-CN powders

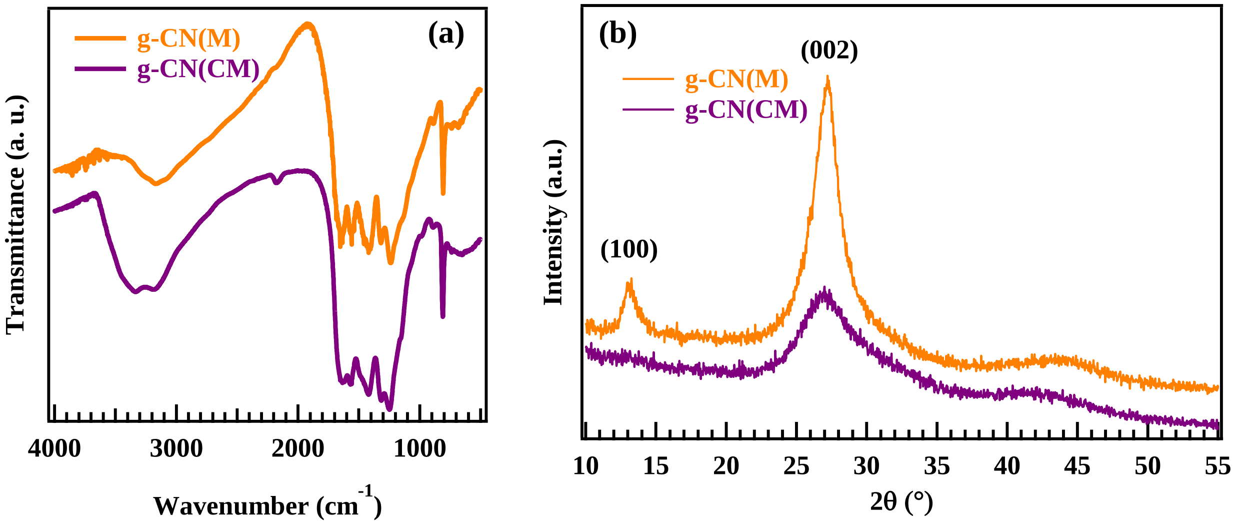

Figure S1. (a) FT-IR spectra, and (b) XRD patterns recorded in the Bragg-Brentano geometry for carbon nitride powders used as EPD precursors.

In the recorded FT-IR spectra (Figure S1a), the broad band between 2900 and 3500 cm^{-1} was assigned to stretching vibrations of –OH groups at the edges of g-CN sheets[21-22] and of primary and secondary amino-groups, whose presence, responsible also for the N-H deformation mode at 885 cm^{-1},[7,11,21,23] was confirmed by X-ray photoelectron spectroscopy (XPS). The higher intensity of the broad band in the 2900-3500 cm^{-1} spectral range for g-CN(CM) in comparison to g-CN(M) suggested that the former was characterized by a higher amount of amino groups. The bands at 810 cm^{-1} and in the interval 1200-1600 cm^{-1} were assigned to breathing modes of triazine units and stretching modes of C-N heterocycles, respectively.[7,11,24]

The XRD pattern of g-CN(M) powders (Figure S1b) was characterized by two signals at 2θ = 13.3 and 27.2°, whereas for g-CN(CM) only a peak at 2θ = 27.0° was observed. The signal at 2θ = 27.0° was ascribed to the interplanar (002) stacking of g-CN sheets.[7,11,25]. The reflection at 2θ = 13.3° for g-CN(M) was attributed to the packing of tri-s-triazine units in (100) crystallographic planes.[23,25] Evaluation of the mean crystallite sizes by means of the Scherrer formula yielded values of (4.0±0.6) nm and (2.0±0.5) nm for g-CN(M) and g-CN(CM), respectively.

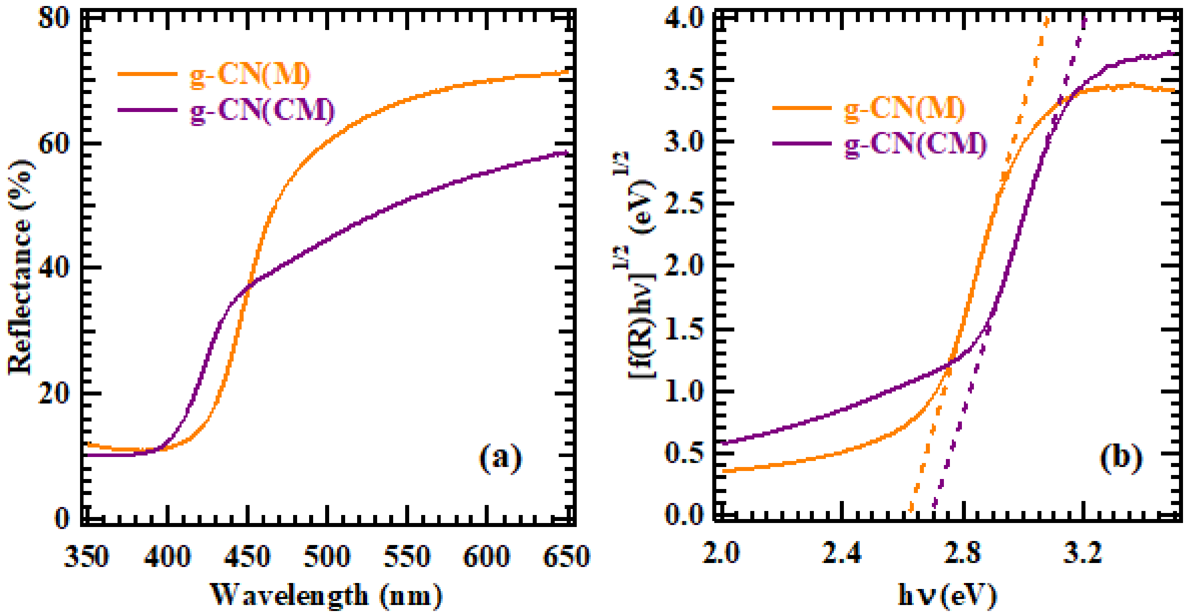

Figure S2. (a) Diffuse reflectance spectra, and (b) Tauc plots for carbon nitride powders. f(R) is the Kubelka-Munk function and R is the measured reflectance-

Optical spectra of g-CN powders (Figure S2a) were characterized by the presence of a net reflectance decrease for $\lambda < 450$ nm, due to g-CN interband electronic transitions. Optical band gaps (E_G), calculated from Tauc plots (Figure S2b), were estimated to be (2.60±0.05) eV and (2.70±0.05) eV for g-CN(M) and g-CN(CM), respectively.[26-27] The slightly higher value for the latter could be attributed to quantum confinement effects due, in turn, to the decreased crystallite size.[7-8,12-13,28]

§ S-2.2 Characterization of the obtained systems

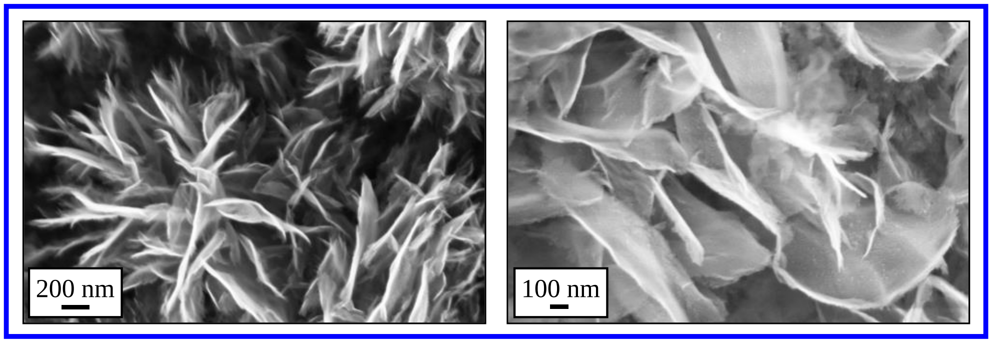

Figure S3. Field emission-scanning electron microscopy (FE-SEM) images at different magnification levels for a bare MnO_2 specimen.

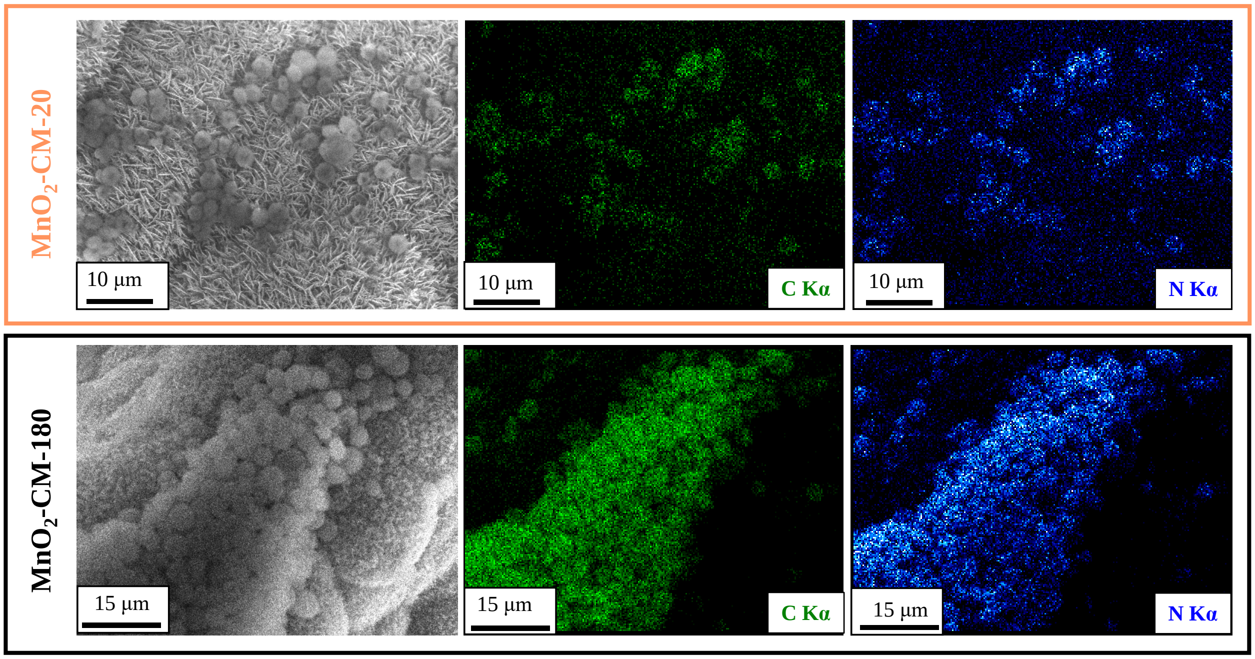

Figure S4. Representative carbon and nitrogen energy dispersive X-ray spectroscopy (EDXS) elemental maps, recorded on the corresponding FE-SEM images, for two MnO_2+g-CN(CM) specimens.

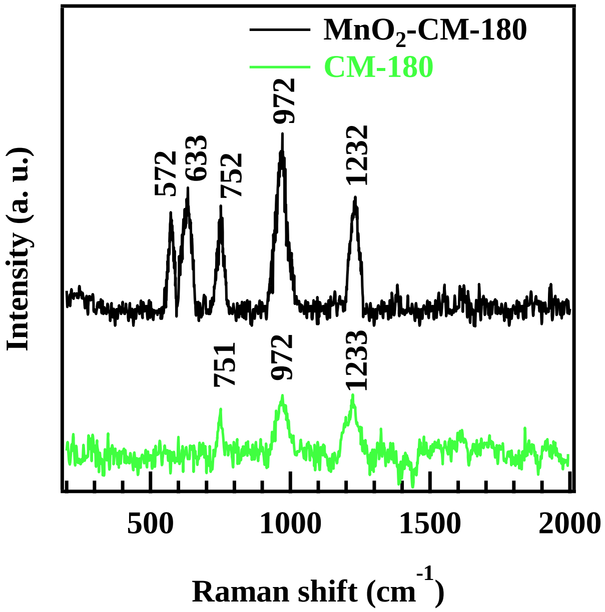

Figure S5. Raman spectra of MnO_2-CM-180 and CM-180 specimens.

Raman spectra for the different samples displayed qualitatively similar features. Figure S5 reports the spectra of two representative systems. The spectral region comprised between 1250 and 400 cm^{-1} is characteristic of the skeletal vibrations of nitrogen aromatic rings.[29] In particular, the signals at 972 and 750 cm^{-1} can be assigned to the breathing mode of heterocyclic heptazine/triazine units[30] and C−N−C bending vibrations, respectively.[31-32] The peak at 1232 cm^{-1} is typical of melon-like g-CN, formed by *zig-zag* chains of heptazine rings linked via –NH– units and laterally terminated by $–NH_2$ groups, linked by H-bonds to yield layers.[33] For specimen MnO_2-CM-180, the two additional signals at 572 and 633 cm^{-1} are attributed to Mn-O stretching vibrations of MnO_6 octahedra in manganese dioxide lattice.[32,34-36]

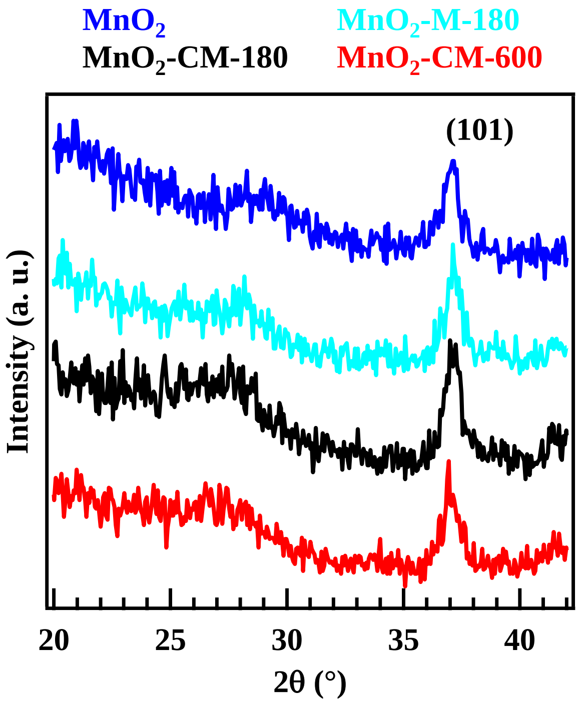

Figure S6. XRD patterns recorded in glancing incidence mode (θ_i = 2.0°) for representative specimens deposited on Ni foams.

XRD patterns of both pristine and functionalized MnO_2 samples (Figure S6) display one well-detectable peak at 2θ = 37.2°, attributable to (101) planes of tetragonal β-MnO_2, the equilibrium phase of manganese(IV) oxide at standard pressure and temperature.[37-38] The weak and very broad reflection at 2θ = 28.5° can be ascribed to the β-MnO_2 (110) signal,[39] and, for composite systems, also to a further contribution from the stacking of (002) carbon nitride graphitic layers.[7,11,25] In this regard, the weak diffracted intensity of g-CN cannot be traced back to the low mass of the functionalizing agent (see the deposited mass values in Figure 1, center bottom panel, of the main paper), but rather to its inherent structural disorder and defectivity, in line also with XPS data (see the main paper text).

It is worth highlighting that the intensity of the peak at 2θ = 37.2° did not undergo any appreciable variation upon g-CN deposition, indicating that the EPD process preserved the features of the underlying β-MnO_2 matrix.

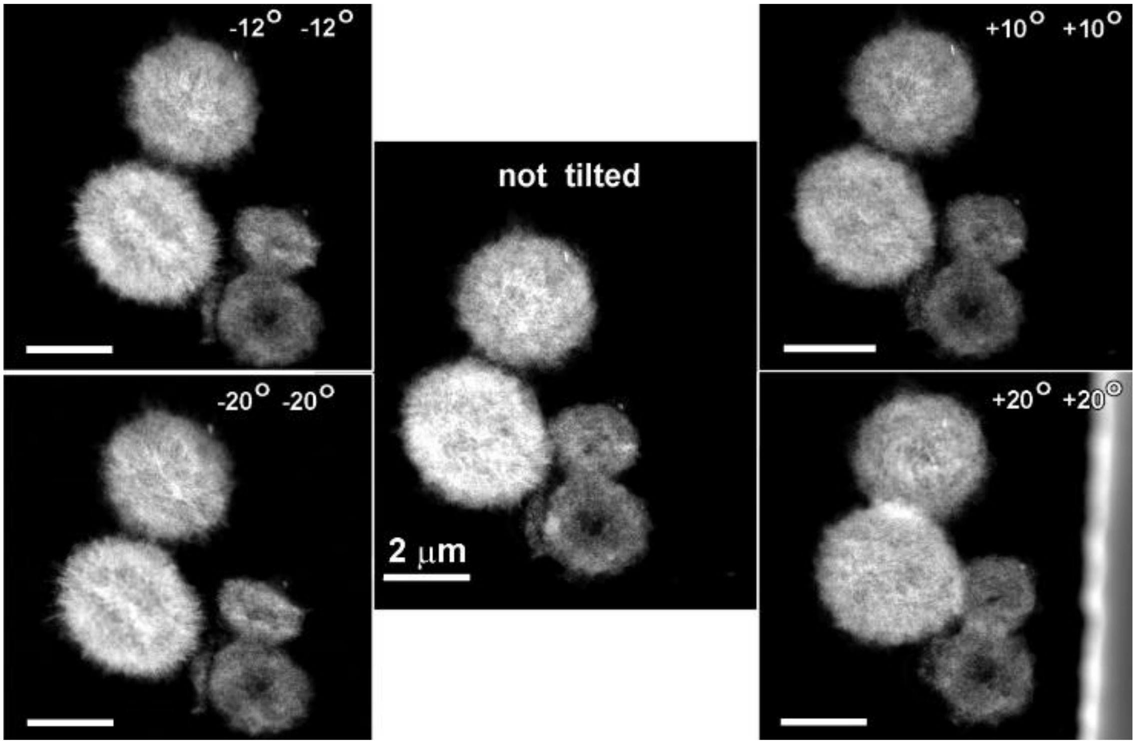

Figure S7. High angle annular dark field-scanning TEM (HAADF-STEM) images of spherical mesoporous and donut-type g-CN particles for specimen MnO_2-CM-45. The scale bar length corresponds to 2 μm in all cases. The orthogonal tilting angles are indicated in each image; for the central image, no tilting has been applied. Such tilting experiments unambiguously confirm the spherical (3D) morphology of g-CN particles.

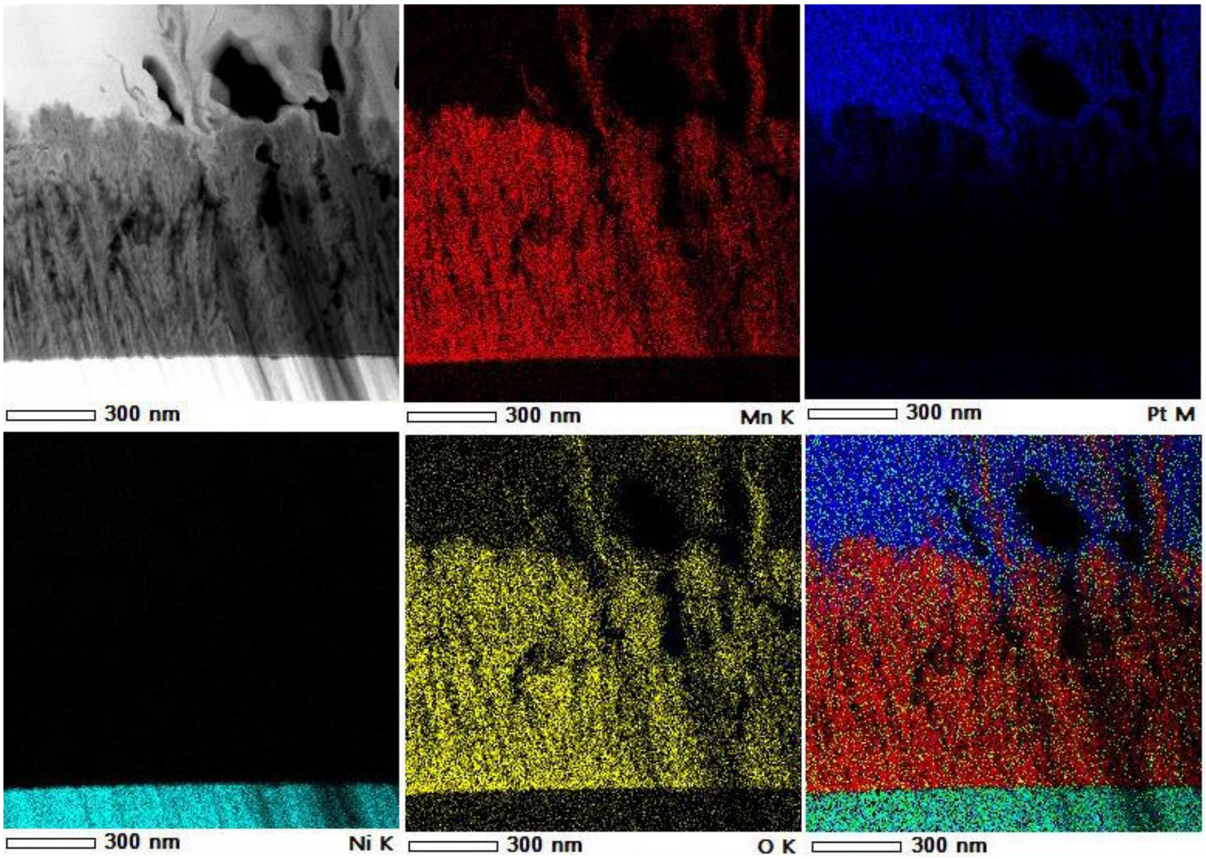

Figure S8. Low magnification HAADF-STEM image and corresponding STEM-EDXS elemental maps for specimen MnO_2-M-180. The Pt signal is due to the protective layer deposited during FIB sample preparation. The right figure in the bottom panel displays a superimposed color image.

Sample	C_0		C_1		C_2	
	**BE (C_0) (eV)**	**% (C_0)**	**BE (C_1) (eV)**	**% (C_1)**	**BE (C_2) (eV)**	**% (C_2)**
**M-180**	284.8	73	286.2	7	288.2	20
**MnO_2-M-180**	284.8	67	286.4	11	288.4	22
**CM-45**	284.8	20	286.2	20	288.3	60
**MnO_2-CM-45**	284.8	18	286.6	22	288.7	60

Table S1. BE (eV) and % contribution of the various C1s components with respect to the overall C1s peak for g-CN and composite specimens supported on Ni foams. Color codes as in Figure 6c. Uncertainty on BE values: ± 0.1 eV; uncertainty on % values: ± 1%.

Sample	N_1		N_2		N_3		N_4	
	**BE (N_1) (eV)**	**% (N_1)**	**BE (N_2) (eV)**	**% (N_2)**	**BE (N_3) (eV)**	**% (N_3)**	**BE (N_4) (eV)**	**% (N_4)**
**M-180**	398.6	70	399.9	19	401.2	6	404.2	5
**MnO_2-M-180**	398.8	69	400.1	16	401.4	10	404.4	5
**CM-45**	398.7	54	399.9	27	401.2	15	404.2	4
**MnO_2-CM-45**	399.1	55	400.3	21	401.6	20	404.6	4

Table S2. BE (eV) and % contribution of the various N1s components with respect to the overall N1s peak for g-CN and composite samples supported on Ni foams. Color codes as in Figure 6c. Uncertainty on BE values: ± 0.1 eV; uncertainty on % values: ± 1%.

Sample	BE ($Mn2p_{3/2}$) (eV)	BE ($Mn2p_{3/2}$) (eV)	SOS (eV)
**MnO_2**	642.6	654.2	11.6
**MnO_2-M-180**	642.4	654.0	11.6
**MnO_2-CM-45**	642.2	653.8	11.6

Table S3. BE (eV) of Mn2p spin-orbit components for the target specimens. SOS = spin-orbit splitting. Uncertainty on BE values: ± 0.1 eV.

Sample	BE [Mn3s(A)] (eV)	BE [Mn3s(B)] (eV)	MS (eV)
**MnO_2**	84.4	89.2	4.8
**MnO_2-M-180**	84.2	89.0	4.8
**MnO_2-CM-45**	84.0	88.8	4.8

Table S4. BE (eV) of Mn3s components for the target specimens. MS = multiplet splitting separation. Uncertainty on BE values: ± 0.1 eV.

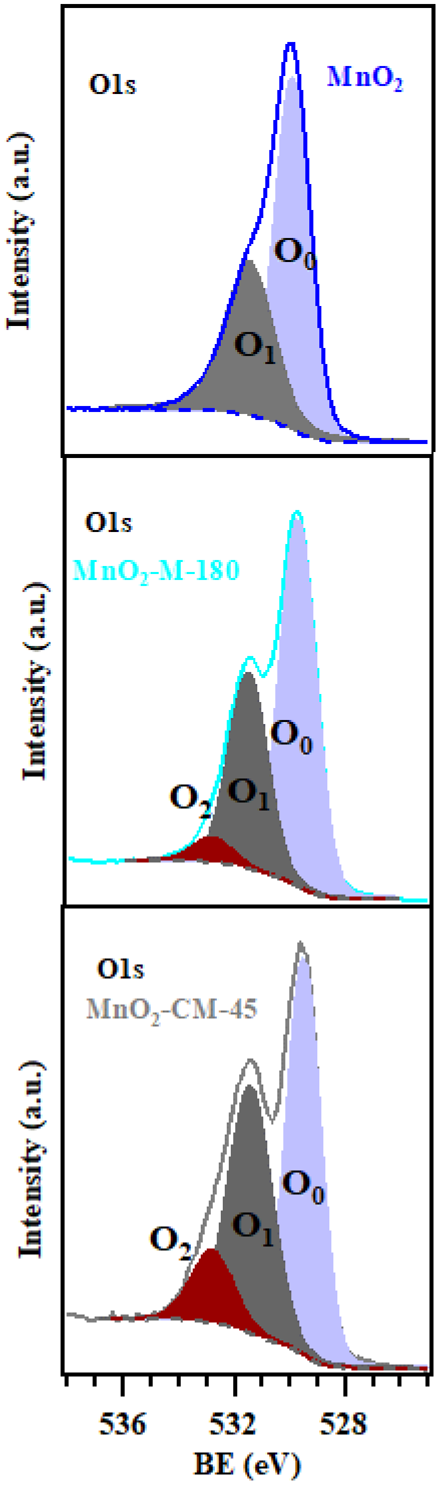

Figure S9. Surface O1s photopeaks for representative MnO_2, carbon nitride, and composite specimens supported on Ni foams.

Sample	O_0		O_1		O_2	
	BE (O_0) (eV)	% (O_0)	BE (O_1) (eV)	% (O_1)	BE (O_2) (eV)	% (O_2)
**MnO_2**	529.9	60	531.5	40	-	-
**M-180**	529.6	40	531.4	60	-	-
**MnO_2-M-180**	529.7	60	531.5	35	532.8	5
**CM-45**	529.6	51	531.4	49	-	-
**MnO_2-CM-45**	529.5	48	531.5	40	532.8	12

Table S5. % contribution of the various O1s components to the overall O1s peak for representative MnO_2, carbon nitride, and composite specimens supported on Ni foams. The average difference between BE ($Mn2p_{3/2}$) (see Table S3) and component **O_0** of the O1s signal always yielded a mean value of 112.7 eV, as expected for MnO_2.[40-41] Uncertainty on BE values: ± 0.1 eV; uncertainty on % values: ± 1%.

The results obtained by O1s peak fitting are displayed in Figure S9 and summarized in Table S5. For specimens M-180 and CM-45, that featured an incomplete substrate coverage, band **O_0** (BE = 529.6 eV) could be related to NiO from the Ni foam.[42] As concerns manganese dioxide-containing samples, band **O_0**, due to lattice oxygen in MnO_2,[40,43-46] underwent a negative BE shift for MnO_2-M-180 (-0.2 eV) and MnO_2-CM-45 (-0.4 eV) if compared to the pristine MnO_2 (for which BE = 529.9 eV, in line with previous studies.[47-48]) The more marked shift for MnO_2-CM-45 was in line with the results obtained by the analysis of Mn2p and Mn3s signals (see the main paper text).

Component **O_1** was assigned to -OH groups adsorbed on oxygen surface vacancies,[34,47-51] confirming the obtainment of defective specimens – an important issue to achieve favorable functional performances.[34] For composite specimens (MnO_2-M-180 and MnO_2-CM-45), the third band (**O_2**, BE = 532.8 eV) was assigned to the formation of C-O-Mn linkages, confirming that carbon nitride was chemically anchored to the underlying MnO_2 deposits.[44-47] The weight of band **O_2** underwent a nearly three-fold enhancement upon going from MnO_2-M-180 to MnO_2-CM-45 (Table S5), indicating the presence of a higher number of C-O-Mn bonds in the latter case. This phenomenon, along with the more marked BE shifts of Mn and lattice O peaks observed for MnO_2-

CM-45 in comparison to MnO_2-M-180, evidenced the occurrence of a more effective heterojunction for MnO_2-CM-45 (see also the main paper text and Figure 11a). Taken together, the present outcomes account for the higher functional performances of g-CN(CM)-containing samples, as indeed observed.

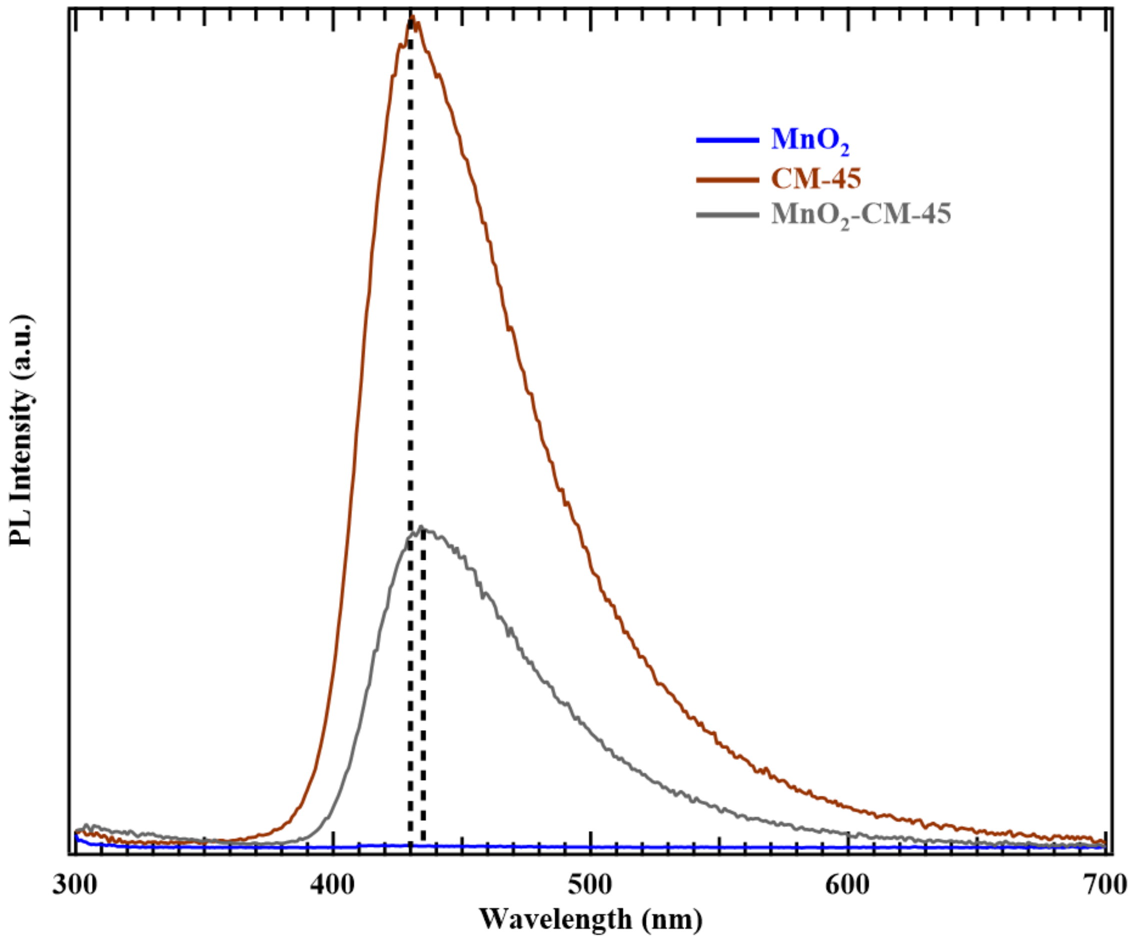

Figure S10. Photoluminescence spectra obtained from bare MnO_2, CM-45 and MnO_2-CM-45 samples. On the basis of previous works,[52-53] the PL spectrum results from two contributions, *i.e.* a first emission at λ = 405 nm, corresponding to the δ^* conduction band → carbon nitride lone pair (LP) transition, and a second one at λ = 480 nm, attributed to the π^* conduction band → LP transition. The relative contribution of these two signals directly influences the overall profile.

§ S-3. Electrochemical tests and related analyses

A valuable estimation of the flat-band potential (E_{fb}) can be obtained as follows,[54] assuming a normal incident photon flux $q_{p,0}(\lambda)$. Photons passing through the semiconductor are progressively adsorbed so that, after a distance x from the interface, there are $q_{p,0}(\lambda)e^{-\alpha x}$ photons left, where α is the absorption coefficient (m^{-1}). The electron-hole pair formation rate is given by: $g(x) = \alpha q_{p,0}(\lambda)e^{-\alpha x}$. The photocurrent generated within the depletion layer (DL) can be obtained by integrating $g(x)$ over the whole DL width w: $j_{DL} = q\int_0^w g(x)dx = q_e\, q_{p,0}(\lambda)[1 - e^{-\alpha w}]$, where the electron charge is denoted by q_e. Thus, the total photocurrent can be expressed as:

$$j_{tot} = q_e q_{p,0}(\lambda)\left[1 - \frac{e^{-\alpha w}}{(1+\alpha L_p)}\right] + q_e p_0 D_p / L_p \qquad \text{(S5)}$$

where L_p (m) and D_p ($m^2 \cdot s^{-1}$) are the hole diffusion length and diffusion coefficient, respectively, and p_0 is the equilibrium hole density. Upon neglecting the photocurrent contribution due to hole diffusion from the bulk, $j_D = q_e p_0 D_p / L_p$, and assuming $\alpha w \ll 1$, so that $e^x \approx 1 - x$, the previous expression reduces to:

$$j_{tot} = q_e q_{p,0}(\lambda)\left[\alpha w_0 \left(E_{fb} - E\right)^{1/2}\right] \qquad \text{(S6)}$$

where w_0 and E are the space charge width constant ($mV^{-1/2}$) and the applied potential, respectively. Squaring both sides and rearranging equation (S6) leads to:

$$E_{fb} - E = \left(\frac{j_{tot}}{q_e q_{p,0}(\lambda)\alpha w_0}\right)^2 \qquad \text{(S7)}$$

Hence, a plot of $(j_{tot})^2$ against potential intersects the x axis at the flat band potential (E_{fb}).

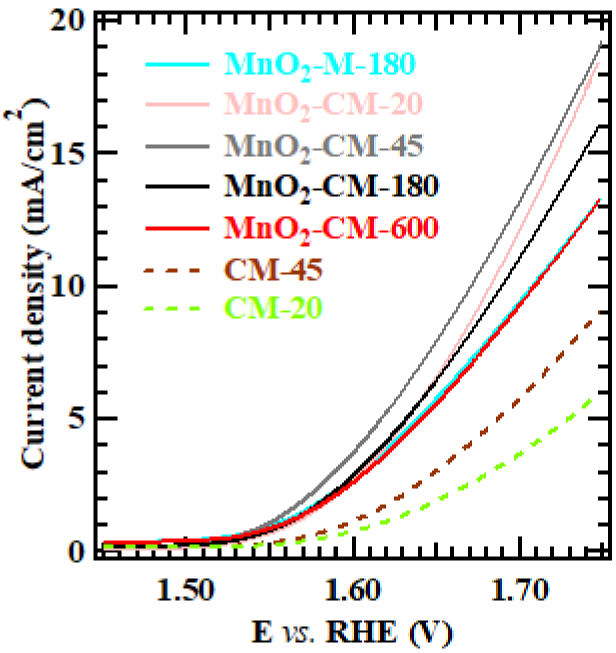

Figure S11. Linear sweep voltammetry (LSV) curves registered in the dark for the indicated specimens.

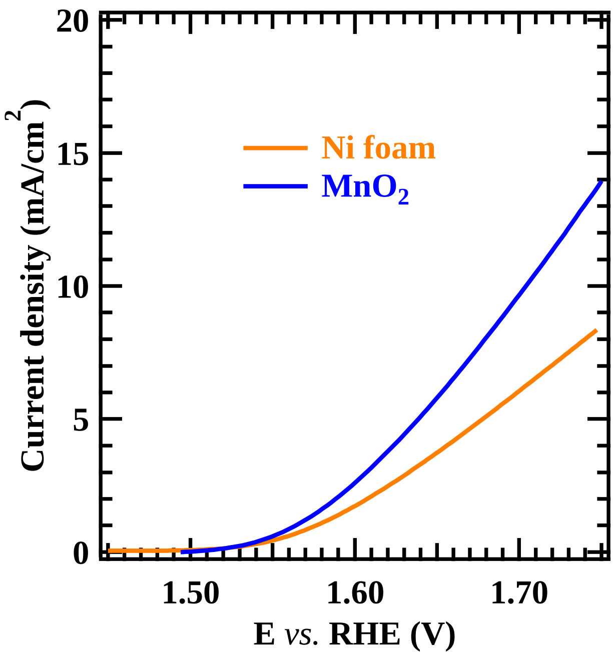

Figure S12. LSV curves for the bare MnO_2 sample, and the uncoated Ni foam substrate, recorded under illumination.

**Material**	**Electrolyte**	**$j_{1.65}$ (mA/cm^2)**	**η @ 10 mA/cm^2 (V *vs.* RHE)**	**TOF × 10^{-3} (s^{-1})**	**E_{Wmax} (V_{RHE})**
MnO_2	0.1 M KOH	5.77	475	3.5	1.13
MnO_2-M-180		5.75	480	3.6	1.11
MnO_2-CM-20		6.97	440	5.2	1.02
MnO_2-CM-45		8.18	430	6.5	1.00
MnO_2-CM-180		6.76	450	4.8	1.04
MnO_2-CM-600		5.95	470	4.4	1.07

Table S6. Representative OER electrochemical performance results for pure MnO_2 and MnO_2+g-CN specimens under illumination: photocurrent densities at E = 1.65 V ($j_{1.65}$) *vs.* RHE, overpotential (η), TOF, and potential of maximum efficiency (E_{Wmax}) corresponding to the maximum in applied bias photon-to-current efficiency (ABPE) curves in Figures 10a.

**Material**	**Electrolyte**	**$j_{1.65}$ (mA/cm^2)**	***η* @ 10 mA/cm^2 (V *vs.* RHE)**	**Tafel slope (mV/ decade)**	**Ref.**
MnO_2	phosphate buffer (pH = 7) 0.07 M	5.0 [a]	460 [b]	105	19
MnO_2@g-C_3N_4		7.0 [a]	420 [b]	90	
MnO_x/C	phosphate buffer (pH = 7) 0.07 M	1.5-3.5 [a]	450 [b]	145-150	55
MnO_2/B,O doped g-C_3N_4	0.1 M KOH	0.50-1.10 [c]	*n.a.*	*n.a.*	34
MnO_2	1.0 M KOH	≈ 21.0 [a]	315	110	35
Co-MnO_2@CNFs		≈ 135.0 [a]	279	75	
MnO_2	0.1 M KOH	≈ 1.1 [a]	630	144	56
MnO_2/CNTs		7.5 [a]	440	67	
MnO_2	1.0 M KOH	≈ 14.0 [a]	370	134	57
MnO_2	1.0 M KOH	≈ 10.0 [a]	420	88	36
MnO_2/CDs	1.0 M KOH	≈ 9.6-13.0 [a]	343-426	44-65	58
MnO_2/CDs	1.0 M KOH	≈ 48.0-56.0 [a]	325-352	73-84	59

Table S7. OER performances of selected electrocatalysts based on MnO_2 and carbon-based materials. [a] dark conditions; [b] @1 mA/cm^2; [c] light conditions. CNFs = carbon nanofibers; CNTs = carbon nanotubes; CDs = carbon dots. *n.a.* = not available.

Material	Electrolyte	$j_{1.65}$ (mA/cm^2)	*η* @ 10 mA/cm^2 (V *vs*. RHE)	Tafel slope (mV/decade)	Ref.
**IrO_2**	1.0 M KOH	18.0	391	149	[60]
**IrO_2**	1.0 M KOH	53.0	321	91	[61]
**IrO_2**	0.1 M KOH	8.0	461	113	[62]
**IrO_2**	1.0 M KOH	27.0	351	67	[63]
**RuO_2**		15.0	371	89	
**RuO_2**	0.1 M KOH	17.0	391	71	[64]
**RuO_2**	1.0 M KOH	13.0	411	74	[65]

Table S8. OER performances of selected IrO_2 and RuO_2 electrocatalysts operating in alkaline media.

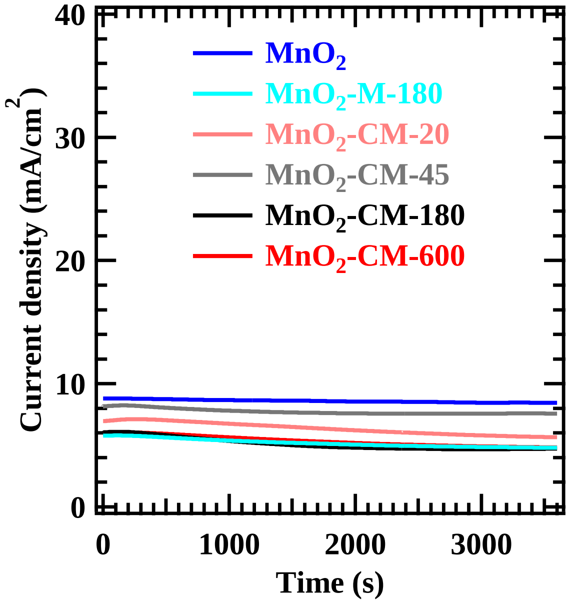

Figure S13. CA measurements for MnO_2 and MnO_2+g-CN electrodes, registered at 1.65 V *vs.* RHE.

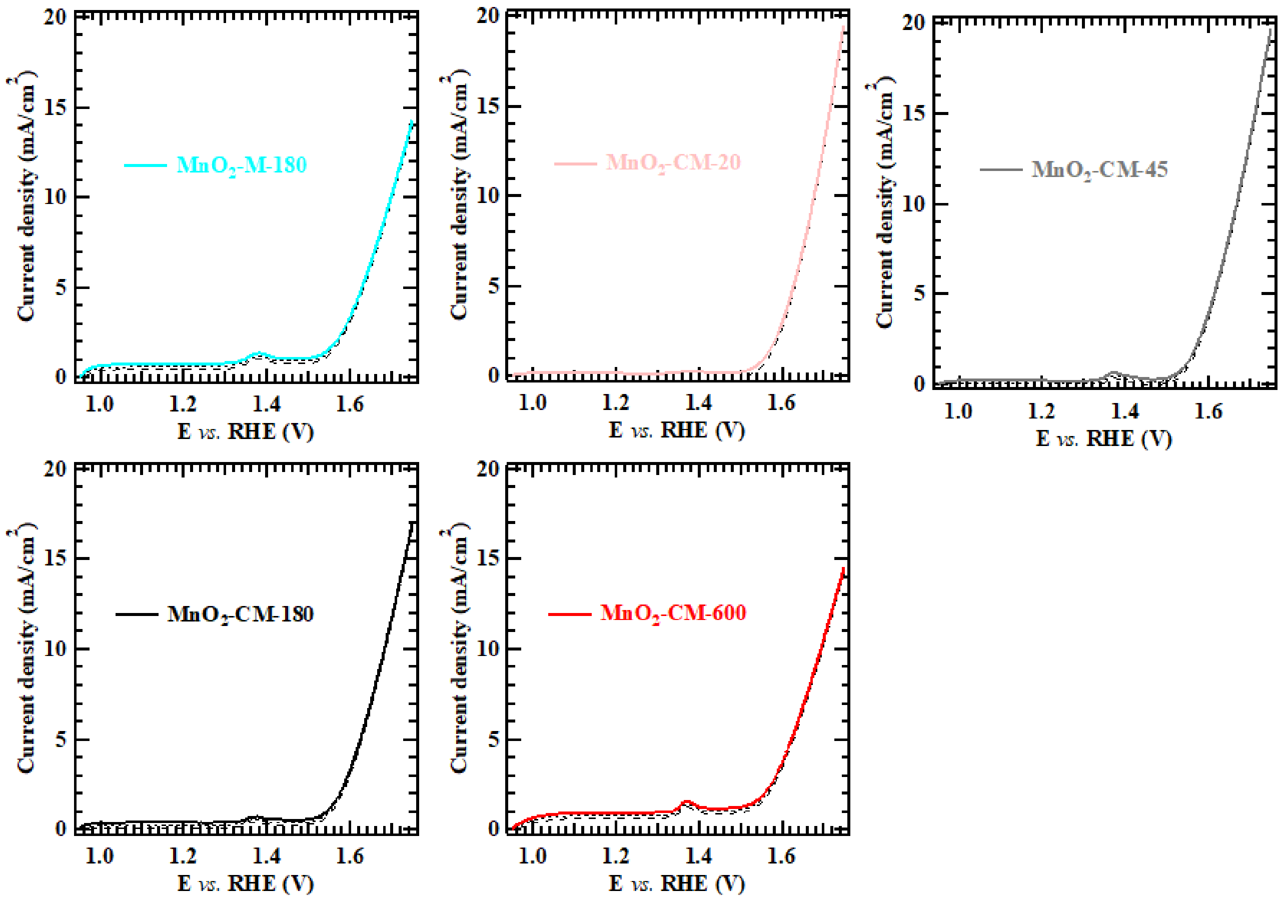

Figure S14. LSV curves recorded on as-prepared specimens (solid lines) and collected every 90 days for six months upon sample storage under ambient conditions (dashed lines).

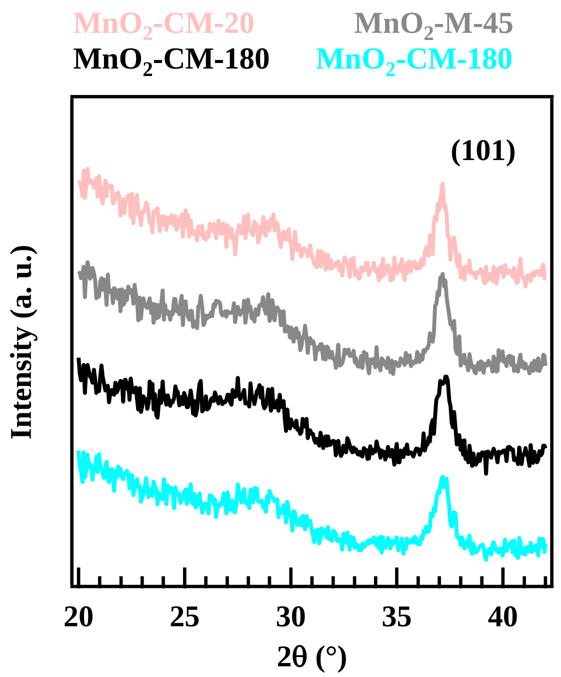

Figure S15. XRD patterns collected in glancing incidence mode ($\theta_i = 2.0°$) after storing specimens under ambient conditions for six months, during which they were electrochemically tested every 90 days.

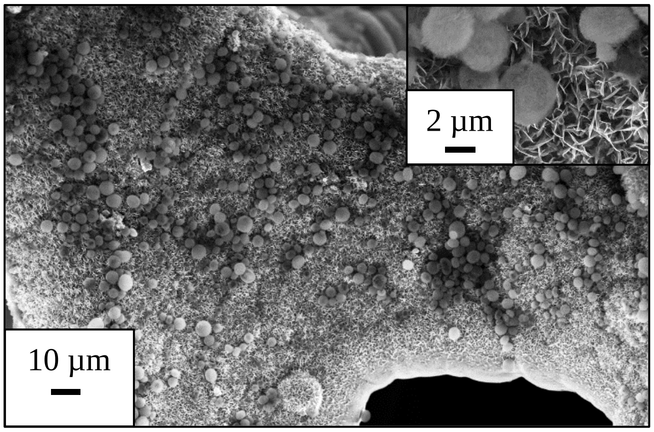

Figure S16. Representative FE-SEM image of MnO_2-CM-45 after storing the specimen under ambient conditions for 6 months, during which the sample was electrochemically tested every 90 days.

XRD measurements on Ni foam-supported MnO_2+g-CN samples after OER tests (Figure S15) revealed that the deposit structural features were preserved after electrochemical tests, indicating a good operational stability. In a similar way, FE-SEM analyses (see Figure S16) indicated that the morphology of composite systems did not undergo any major alteration.

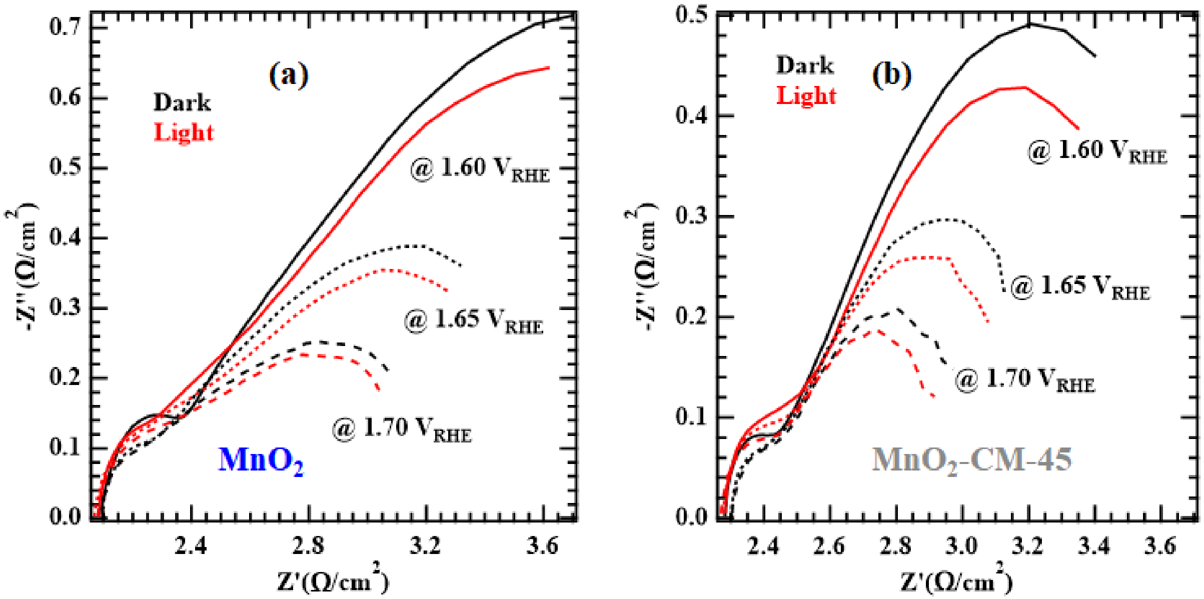

Figure S17. EIS spectra, reported as Nyquist plots, for specimens MnO_2 (a) and MnO_2-CM-45 (b). The lower charge transfer resistance under illumination in the case of sample MnO_2-CM-45 is an indication of an effective MnO_2/g-CN junction, leading to a better charge carrier separation.

§ S-4. References

(1) Urbain, F.; Du, R.; Tang, P.; Smirnov, V.; Andreu, T.; Finger, F.; Jimenez Divins, N.; Llorca, J.; Arbiol, J.; Cabot, A.; Morante, J. R. Upscaling High Activity Oxygen Evolution Catalysts Based on $CoFe_2O_4$ Nanoparticles Supported on Nickel Foam for Power-to-Gas Electrochemical Conversion with Energy Efficiencies above 80%. *Appl. Catal., B* **2019**, *259*, 118055.

(2) Xiao, C.; Li, S.; Zhang, X.; MacFarlane, D. R. $MnO_2/MnCo_2O_4/Ni$ Heterostructure with Quadruple Hierarchy: a Bifunctional Electrode Architecture for Overall Urea Oxidation. *J. Mater. Chem. A* **2017**, *5*, 7825-7832.

(3) Chaudhari, N. K.; Jin, H.; Kim, B.; Lee, K. Nanostructured Materials on 3D Nickel Foam as Electrocatalysts for Water Splitting. *Nanoscale* **2017**, *9*, 12231-12247.

(4) Bigiani, L.; Andreu, T.; Maccato, C.; Fois, E.; Gasparotto, A.; Sada, C.; Tabacchi, G.; Krishnan, D.; Verbeeck, J.; Morante, J. R.; Barreca, D. Engineering Au/MnO_2 Hierarchical Nanoarchitectures for Ethanol Electrochemical Valorization. *J. Mater. Chem. A* **2020**, *8*, 16902-16907.

(5) Barreca, D.; Carraro, G.; Fois, E.; Gasparotto, A.; Gri, F.; Seraglia, R.; Wilken, M.; Venzo, A.; Devi, A.; Tabacchi, G.; Maccato, C. Manganese(II) Molecular Sources for Plasma-Assisted CVD of Mn Oxides and Fluorides: From Precursors to Growth Process. *J. Phys. Chem. C* **2018**, *122*, 1367-1375.

(6) Maccato, C.; Bigiani, L.; Carraro, G.; Gasparotto, A.; Seraglia, R.; Kim, J.; Devi, A.; Tabacchi, G.; Fois, E.; Pace, G.; Di Noto, V.; Barreca, D. Molecular Engineering of Mn^{II} Diamine Diketonate Precursors for the Vapor Deposition of Manganese Oxide Nanostructures. *Chem. Eur. J.* **2017**, *23*, 17954-17963.

(7) Benedet, M.; Rizzi, G. A.; Gasparotto, A.; Lebedev, O. I.; Girardi, L.; Maccato, C.; Barreca, D. Tailoring Oxygen Evolution Performances of Carbon Nitride Systems Fabricated by Electrophoresis through Ag and Au Plasma Functionalization. *Chem. Eng. J.* **2022**, *448*, 137645.

(8) Jun, Y. S.; Lee, E. Z.; Wang, X.; Hong, W. H.; Stucky, G. D.; Thomas, A. From Melamine-Cyanuric Acid Supramolecular Aggregates to Carbon Nitride Hollow Spheres. *Adv. Funct. Mater.* **2013**, *23*, 3661-3667.

(9) Zhang, S.; Yan, J.; Yang, S.; Xu, Y.; Cai, X.; Li, X.; Zhang, X.; Peng, F.; Fang, Y.

Electrodeposition of Cu_2O/g-C_3N_4 Heterojunction Film on an FTO Substrate for Enhancing Visible Light Photoelectrochemical Water Splitting. *Chin. J. Catal.* **2017**, *38*, 365-371.

(10) Besra, L.; Liu, M. A Review on Fundamentals and Applications of Electrophoretic Deposition (EPD). *Prog. Mater. Sci.* **2007**, *52*, 1-61.

(11) Benedet, M.; Rizzi, G. A.; Gasparotto, A.; Gauquelin, N.; Orekhov, A.; Verbeeck, J.; Maccato, C.; Barreca, D. Functionalization of Graphitic Carbon Nitride Systems by Cobalt and Cobalt-Iron Oxides Boosts Solar Water Oxidation Performances. *Appl. Surf. Sci.* **2023**, *618*, 156652.

(12) Jiang, Y.; Sun, Z.; Tang, C.; Zhou, Y.; Zeng, L.; Huang, L. Enhancement of Photocatalytic Hydrogen Evolution Activity of Porous Oxygen Doped g-C_3N_4 with Nitrogen Defects Induced by Changing Electron Transition. *Appl. Catal., B* **2019**, *240*, 30-38.

(13) Liu, W.; Zhang, Z.; Zhang, D.; Wang, R.; Zhang, Z.; Qiu, S. Synthesis of Narrow-Band Curled Carbon Nitride Nanosheets with High Specific Surface Area for Hydrogen Evolution from Water Splitting by Low-Temperature Aqueous Copolymerization to Form Copolymers. *RSC Adv.* **2020**, *10*, 28848-28855.

(14) Bigiani, L.; Maccato, C.; Andreu, T.; Gasparotto, A.; Sada, C.; Modin, E.; Lebedev, O. I.; Morante, J. R.; Barreca, D. Quasi-1D Mn_2O_3 Nanostructures Functionalized with First-Row Transition-Metal Oxides as Oxygen Evolution Catalysts. *ACS Appl. Nano Mater.* **2020**, *3*, 9889-9898.

(15) McCrory, C. C. L.; Jung, S.; Peters, J. C.; Jaramillo, T. F. Benchmarking Heterogeneous Electrocatalysts for the Oxygen Evolution Reaction. *J. Am. Chem. Soc.* **2013**, *135*, 16977-16987.

(16) Connor, P.; Schuch, J.; Kaiser, B.; Jaegermann, W. The Determination of Electrochemical Active Surface Area and Specific Capacity Revisited for the System MnO_x as an Oxygen Evolution Catalyst. *Z. Phys. Chem.* **2020**, *234*, 979.

(17) Bigiani, L.; Barreca, D.; Gasparotto, A.; Andreu, T.; Verbeeck, J.; Sada, C.; Modin, E.; Lebedev, O. I.; Morante, J. R.; Maccato, C. Selective Anodes for Seawater Splitting via Functionalization of Manganese Oxides by a Plasma-Assisted Process. *Appl. Catal., B* **2021**, *284*, 119684.

(18) Maccato, C.; Bigiani, L.; Girardi, L.; Gasparotto, A.; Lebedev, O. I.; Modin, E.; Barreca,

D.; Rizzi, G. A. Plasma-Assisted Synthesis of Co_3O_4-Based Electrocatalysts on Ni Foam Substrates for the Oxygen Evolution Reaction. *Adv. Mater. Interfaces* **2021**, *8*, 2100763.

(19) Elmacı, G.; Ertürk, A. S.; Sevim, M.; Metin, Ö. MnO_2 Nanowires Anchored on Mesoporous Graphitic Carbon Nitride (MnO_2@mpg-C_3N_4) as a Highly Efficient Electrocatalyst for the Oxygen Evolution Reaction. *Int. J. Hydrogen Energy* **2019**, *44*, 17995-18006.

(20) Song, F.; Hu, X. Ultrathin Cobalt–Manganese Layered Double Hydroxide Is an Efficient Oxygen Evolution Catalyst. *J. Am. Chem. Soc.* **2014**, *136*, 16481-16484.

(21) Görmez, Ö.; Yakar, E.; Gözmen, B.; Kayan, B.; Khataee, A. $CoFe_2O_4$ Nanoparticles Decorated onto Graphene Oxide and Graphitic Carbon Nitride layers as a Separable Catalyst for Ultrasound-Assisted Photocatalytic Degradation of Bisphenol-A. *Chemosphere* **2022**, *288*, 132663.

(22) Zhou, L.; Lei, J.; Wang, F.; Wang, L.; Hoffmann, M. R.; Liu, Y.; In, S.-I.; Zhang, J. Carbon Nitride Nanotubes with In Situ Grafted Hydroxyl Groups for Highly Efficient Spontaneous H_2O_2 Production. *Appl. Catal., B* **2021**, *288*, 119993.

(23) Kessler, F. K.; Zheng, Y.; Schwarz, D.; Merschjann, C.; Schnick, W.; Wang, X.; Bojdys, M. J. Functional Carbon Nitride Materials - Design Strategies for Electrochemical Devices. *Nat. Rev. Mater.* **2017**, *2*, 17030.

(24) Yuan, X.; Luo, K.; Zhang, K.; He, J.; Zhao, Y.; Yu, D. Combinatorial Vibration-Mode Assignment for the FTIR Spectrum of Crystalline Melamine: A Strategic Approach Toward Theoretical IR Vibrational Calculations of Triazine-Based Compounds. *J. Phys. Chem. A* **2016**, *120*, 7427-7433.

(25) Fina, F.; Callear, S. K.; Carins, G. M.; Irvine, J. T. S. Structural Investigation of Graphitic Carbon Nitride via XRD and Neutron Diffraction. *Chem. Mater.* **2015**, *27*, 2612-2618.

(26) Wang, X.; Maeda, K.; Thomas, A.; Takanabe, K.; Xin, G.; Carlsson, J. M.; Domen, K.; Antonietti, M. A Metal-Free Polymeric Photocatalyst for Hydrogen Production from Water under Visible Light. *Nat. Mater.* **2009**, *8*, 76-80.

(27) Ragupathi, V.; Panigrahi, P.; Ganapathi Subramaniam, N. Bandgap Engineering in Graphitic Carbon Nitride: Effect of Precursors. *Optik* **2020**, *202*, 163601.

(28) Gu, Z.; Asakura, Y.; Yin, S. High Yield Post-Thermal Treatment of Bulk Graphitic Carbon Nitride with Tunable Band Structure for Enhanced deNO_x Photocatalysis. *Nanotechnol.*

2020, *31*, 114001.

(29) Praus, P.; Svoboda, L.; Ritz, M.; Troppová, I.; Šihor, M.; Kočí, K. Graphitic Carbon Nitride: Synthesis, Characterization and Photocatalytic Decomposition of Nitrous Oxide. *Mater. Chem. Phys.* **2017**, *193*, 438-446.

(30) Lin, L.; Ou, H.; Zhang, Y.; Wang, X. Tri-s-triazine-Based Crystalline Graphitic Carbon Nitrides for Highly Efficient Hydrogen Evolution Photocatalysis. *ACS Catal.* **2016**, *6*, 3921-3931.

(31) Lei, G.; Cao, Y.; Zhao, W.; Dai, Z.; Shen, L.; Xiao, Y.; Jiang, L. Exfoliation of Graphitic Carbon Nitride for Enhanced Oxidative Desulfurization: A Facile and General Strategy. *ACS Sustainable Chem. Eng.* **2019**, *7*, 4941-4950.

(32) Soltani, H.; Bahiraei, H.; Ghasemi, S. Effect of Electrodeposition Time on the Super-Capacitive Performance of Electrodeposited MnO_2 on g-C_3N_4 nanosheets. *J. Alloys Compd.* **2022**, *904*, 163565.

(33) Miller, T. S.; Jorge, A. B.; Suter, T. M.; Sella, A.; Corà, F.; McMillan, P. F. Carbon Nitrides: Synthesis and Characterization of a New Class of Functional Materials. *Phys. Chem. Chem. Phys* **2017**, *19*, 15613-15638.

(34) Mishra, B. P.; Acharya, L.; Subudhi, S.; Parida, K. Oxygen Vacancy Rich α-MnO_2 @B/O-g-C_3N_4 Photocatalyst: A Thriving 1D-2D Surface Interaction Effective Towards Photocatalytic O_2 and H_2 Evolution Through Z-Scheme Charge Dynamics. *Int. J. Hydrogen Energy* **2022**, *47*, 32107-32120.

(35) Zhao, Y.; Zhang, J.; Wu, W.; Guo, X.; Xiong, P.; Liu, H.; Wang, G. Cobalt-Doped MnO_2 Ultrathin Nanosheets with Abundant Oxygen Vacancies Supported on Functionalized Carbon Nanofibers for Efficient Oxygen Evolution. *Nano Energy* **2018**, *54*, 129-137.

(36) Samal, R.; Kandasamy, M.; Chakraborty, B.; Rout, C. S. Experimental and Theoretical Realization of an Advanced Bifunctional 2D δ-MnO_2 Electrode for Supercapacitor and Oxygen Evolution Reaction via Defect Engineering. *Int. J. Hydrogen Energy* **2021**, *46*, 28028-28042.

(37) Islam, S.; Alfaruqi, M. H.; Mathew, V.; Song, J.; Kim, S.; Kim, S.; Jo, J.; Baboo, J. P.; Pham, D. T.; Putro, D. Y.; Sun, Y.-K.; Kim, J. Facile Synthesis and the Exploration of the Zinc Storage Mechanism of β-MnO_2 Nanorods with Exposed (101) Planes as a Novel Cathode Material for High Performance Eco-Friendly Zinc-Ion Batteries. *J. Mater. Chem.*

A **2017**, *5*, 23299-23309.

(38) Cheng, F.; Zhang, T.; Zhang, Y.; Du, J.; Han, X.; Chen, J. Enhancing Electrocatalytic Oxygen Reduction on MnO_2 with Vacancies. *Angew. Chem. Int. Ed.* **2013**, *52*, 2474-2477.

(39) Pattern N° 024-0735, JCPDS (2000).

(40) Bigiani, L.; Maccato, C.; Barreca, D.; Gasparotto, A. MnO_2 Nanomaterials Functionalized with Ag and SnO_2: An XPS Study. *Surf. Sci. Spectra* **2020**, *27*, 024005.

(41) Mattelaer, F.; Bosserez, T.; Ronge, J.; Martens, J. A.; Dendooven, J.; Detavernier, C. Manganese Oxide Films with Controlled Oxidation State for Water Splitting Devices through a Combination of Atomic Layer Deposition and Post-Deposition Annealing. *RSC Adv.* **2016**, *6*, 98337-98343.

(42) http://srdata.nist.gov/xps.

(43) Gri, F.; Bigiani, L.; Gasparotto, A.; Maccato, C.; Barreca, D. XPS Investigation of F-doped MnO_2 Nanosystems Fabricated by Plasma Assisted-CVD. *Surf. Sci. Spectra* **2018**, *25*, 024004.

(44) Shi, Y.; Zhang, M.; Li, Y.; Liu, G.; Jin, R.; Wang, Q.; Xu, H.; Gao, S. 2D/1D Protonated g-C_3N_4/α-MnO_2 Z-Scheme Heterojunction with Enhanced Visible-Light Photocatalytic Efficiency. *Ceram. Int.* **2020**, *46*, 25905-25914.

(45) Shi, Y.; Gao, S.; Yuan, Y.; Liu, G.; Jin, R.; Wang, Q.; Xu, H.; Lu, J. Rooting MnO_2 into Protonated g-C_3N_4 by Intermolecular Hydrogen Bonding for Endurable Supercapacitance. *Nano Energy* **2020**, *77*, 105153.

(46) Li, X.; Fang, G.; Qian, X.; Tian, Q. Z-Scheme Heterojunction of Low Conduction Band Potential MnO_2 and Biochar-Based g-C_3N_4 for Efficient Formaldehyde Degradation. *Chem. Eng. J.* **2022**, *428*, 131052.

(47) Xia, P.; Zhu, B.; Cheng, B.; Yu, J.; Xu, J. 2D/2D g-C_3N_4/MnO_2 Nanocomposite as a Direct Z-Scheme Photocatalyst for Enhanced Photocatalytic Activity. *ACS Sustainable Chem. Eng.* **2018**, *6*, 965-973.

(48) Zhang, Y.; Li, H.; Zhang, L.; Gao, R.; Dai, W.-L. Construction of Highly Efficient 3D/2D MnO_2/g-C_3N_4 Nanocomposite in the Epoxidation of Styrene with TBHP. *ACS Sustainable Chem. Eng.* **2019**, *7*, 17008-17019.

(49) Pan, X.; Kong, F.; Xing, M. Spatial Separation of Photo-Generated Carriers in g-C_3N_4/MnO_2/Pt with Enhanced H_2 Evolution and Organic Pollutant Control. *Res. Chem.*

Intermed. **2022**, *48*, 2837-2855.

(50) Bigiani, L.; Gasparotto, A.; Maccato, C.; Sada, C.; Verbeeck, J.; Andreu, T.; Morante, J. R.; Barreca, D. Dual Improvement of β-MnO_2 Oxygen Evolution Electrocatalysts via Combined Substrate Control and Surface Engineering. *ChemCatChem* **2020**, *12*, 5984-5992.

(51) Barreca, D.; Gri, F.; Gasparotto, A.; Carraro, G.; Bigiani, L.; Altantzis, T.; Žener, B.; Lavrenčič Štangar, U.; Alessi, B.; Padmanaban, D. B.; Mariotti, D.; Maccato, C. Multi-functional MnO_2 nanomaterials for photo-activated applications by a plasma-assisted fabrication route. *Nanoscale* **2019**, *11*, 98-108.

(52) Zhang, Y.; Pan, Q.; Chai, G.; Liang, M.; Dong, G.; Zhang, Q.; Qiu, J. Synthesis and Luminescence Mechanism of Multicolor-Emitting g-C_3N_4 Nanopowders by Low Temperature Thermal Condensation of Melamine. *Sci. Rep.* **2013**, *3*, 1943.

(53) Panimalar, S.; Uthrakumar, R.; Selvi, E. T.; Gomathy, P.; Inmozhi, C.; Kaviyarasu, K.; Kennedy, J. Studies of MnO_2/g-C_3N_4 Hetrostructure Efficient of Visible Light Photocatalyst for Pollutants Degradation by Sol-Gel Technique. *Surf. Interfaces* **2020**, *20*, 100512.

(54) Alexander, J. C. *Surface Modifications and Growth of Titanium Dioxide for Photo-Electrochemical Water Splitting*. Springer Theses: 2016.

(55) Melder, J.; Kwong, W. L.; Shevela, D.; Messinger, J.; Kurz, P. Electrocatalytic Water Oxidation by MnO_x/C: In Situ Catalyst Formation, Carbon Substrate Variations, and Direct O_2/CO_2 Monitoring by Membrane-Inlet Mass Spectrometry. *ChemSusChem* **2017**, *10*, 4491-4502.

(56) Xu, N.; Nie, Q.; Luo, L.; Yao, C.; Gong, Q.; Liu, Y.; Zhou, X.-D.; Qiao, J. Controllable Hortensia-Like MnO_2 Synergized with Carbon Nanotubes as an Efficient Electrocatalyst for Long-Term Metal–Air Batteries. *ACS Appl. Mater. Interfaces* **2019**, *11*, 578-587.

(57) Zhou, Y.; Chen, F.; Tian, R.; Huang, S.; Chen, R.; Li, M.; Wan, T.; Han, Z.; Wang, D.; Chu, D. Oxygen Vacancies and Band Gap Engineering of Vertically Aligned MnO_2 Porous Nanosheets for Efficient Oxygen Evolution Reaction. *Surf. Interfaces* **2021**, *26*, 101398.

(58) Tian, L.; Wang, J.; Wang, K.; Wo, H.; Wang, X.; Zhuang, W.; Li, T.; Du, X. Carbon-Quantum-Dots-Embedded MnO_2 Nanoflower as an Efficient Electrocatalyst for Oxygen Evolution in Alkaline Media. *Carbon* **2019**, *143*, 457-466.

(59) Tian, L.; Zhai, X.; Wang, X.; Pang, X.; Li, J.; Li, Z. Morphology and Phase Transformation of α-MnO_2/MnOOH Modulated by N-CDs for Efficient Electrocatalytic Oxygen Evolution Reaction in Alkaline Medium. *Electrochim. Acta* **2020**, *337*, 135823.

(60) Jiao, L.; Zhou, Y.-X.; Jiang, H.-L. Metal–Organic Framework-Based CoP/Reduced Graphene Oxide: High-Performance Bifunctional Electrocatalyst for Overall Water Splitting. *Chem. Sci.* **2016**, *7*, 1690-1695.

(61) Qazi, U. Y.; Yuan, C.-Z.; Ullah, N.; Jiang, Y.-F.; Imran, M.; Zeb, A.; Zhao, S.-J.; Javaid, R.; Xu, A.-W. One-Step Growth of Iron–Nickel Bimetallic Nanoparticles on FeNi Alloy Foils: Highly Efficient Advanced Electrodes for the Oxygen Evolution Reaction. *ACS Appl. Mater. Interfaces* **2017**, *9*, 28627-28634.

(62) Sim, H.; Lee, J.; Yu, T.; Lim, B. Manganese Oxide with Different Composition and Morphology as Electrocatalyst for Oxygen Evolution Reaction. *Korean J. Chem. Eng.* **2018**, *35*, 257-262.

(63) Xiao, Q.; Zhang, Y.; Guo, X.; Jing, L.; Yang, Z.; Xue, Y.; Yan, Y.-M.; Sun, K. A High-Performance Electrocatalyst for Oxygen Evolution Reactions based on Electrochemical Post-Treatment of Ultrathin Carbon Layer Coated Cobalt Nanoparticles. *Chem. Commun.* **2014**, *50*, 13019-13022.

(64) Ahmed, M. S.; Choi, B.; Kim, Y.-B. Development of Highly Active Bifunctional Electrocatalyst using Co_3O_4 on Carbon Nanotubes for Oxygen Reduction and Oxygen Evolution. *Sci. Rep.* **2018**, *8*, 2543.

(65) Das, D.; Das, A.; Reghunath, M.; Nanda, K. K. Phosphine-Free Avenue to Co_2P Nanoparticle Encapsulated N,P Co-Doped CNTs: A Novel Non-Enzymatic Glucose Sensor and an efficient Electrocatalyst for Oxygen Evolution Reaction. *Green Chem.* **2017**, *19*, 1327-1335.
